# FTO-mediated m^6^A demethylation regulates GnRH expression in the hypothalamus via the PLCβ3/Ca^2+^/CAMK signalling pathway

**DOI:** 10.1038/s42003-023-05677-2

**Published:** 2023-12-21

**Authors:** Shaolian Zang, Xiaoqin Yin, Pin Li

**Affiliations:** Department of endocrinology, Shanghai Children’s Hospital, School of medicine, Shanghai Jiao Tong University, 200062 Shanghai, China

**Keywords:** Endocrine reproductive disorders, Gonadal disorders

## Abstract

N^6^-methyladenosine (m^6^A) plays a crucial role in the development and functional homeostasis of the central nervous system. The fat mass and obesity-associated (*FTO*) gene, which is highly expressed in the hypothalamus, is closely related to female pubertal development. In this study, we found that m^6^A methylation decreased in the hypothalamus gradually with puberty and decreased in female rats with precocious puberty. FTO expression was increased at the same time. Methylated RNA immunoprecipitation sequencing (MeRIP-seq) showed that the m^6^A methylation of PLCβ_3_, a key enzyme of the Ca^2+^ signalling pathway, was decreased significantly in the hypothalamus in precocious rats. Upregulating FTO increased PLCβ3 expression and activated the Ca^2+^ signalling pathway, which promoted GnRH expression. Dual-luciferase reporter and MeRIP-qPCR assays confirmed that FTO regulated m^6^A demethylation of PLCβ_3_ and promoted PLCβ_3_ expression. Upon overexpressing FTO in the hypothalamic arcuate nucleus (ARC) in female rats, we observed advanced puberty onset. Meanwhile, PLCβ_3_ and GnRH expression in the hypothalamus increased significantly, and the Ca^2+^ signalling pathway was activated. Our study demonstrates that FTO enhances GnRH expression, which promotes puberty onset, by regulating m^6^A demethylation of PLCβ3 and activating the Ca^2+^ signalling pathway.

## Abstract (web summary)

A study based on in vivo and in vitro experiments suggests that FTO enhances GnRH expression, which promotes puberty onset, by regulating m6A demethylation of PLCβ3 and activating the Ca^2+^ signalling pathway.

## Introduction

Puberty is an essential period of psychological and physical development. During this period, reproductive capacity is achieved. The hallmark of pubertal onset is a reactivation of the hypothalamic–pituitary–gonadal axis (HPGA) with a diurnal increase in pulsatile luteinizing hormone (LH) secretion^1^. This change is driven by an increase in gonadotropin-releasing hormone (GnRH) release from hypothalamic neurons. A primary transsynaptic mechanism underlying pulsatile GnRH release involves KNDy neurons in the arcuate nucleus (ARC) of the hypothalamus^2^. Kisspeptin is a product of the *Kiss1* gene. It acts through the Kiss1 receptor (kiss1r) and is the main “gatekeeper” controlling the onset of puberty, primarily by activating GnRH neurons^3^. Central precocious puberty (CPP) results from premature activation of the HPGA, which leads to the release of GnRH from the hypothalamus in advance^4^. The annual incidence of CPP is increasing gradually, and girls are 5-10 times more likely to develop CPP than boys^5,6^. It is particularly worrying that CPP has some short- and long-term implications for women, including an increased risk of psychosocial distress, short stature, breast cancer, endometrial cancer, obesity, type 2 diabetes, and cardiovascular disease in adulthood^7^. For these reasons, CPP has long been a critical issue in reproductive endocrinology research^8,9^. Thus far, studies on the pathogenesis of CPP have mainly focused on energy metabolism^10^, environmental pollution^11^, the gut microbiome^12^ and genetic inheritance^13–15^. The molecular mechanisms of CPP are only beginning to be understood. Some researchers have proposed that the epigenetic control of puberty onset may play a fundamental role in the initiation of puberty^16^. Many studies have also identified epigenetic processes, including DNA methylation and hydroxymethylation, histone posttranslational modifications and noncoding RNA activities, that affect the reactivation of the hypothalamic GnRH pulse generator around puberty in rodents and primates, such as trimethylation of histone H3 at lysine 27 (H3K27me3) and the activities of lysine demethylase 6b (Kdm6b), miR-200 and miR-155^17–19^. However, it is not known whether RNA methylation is involved in regulating puberty onset.

The most representative RNA methylations are m^6^A and m^5^C^20^. It has been reported that RNA-m^6^A methylation is involved in nervous system development, spermatogenesis, synaptic transmission, synaptic plasticity, neural signalling, and RNA metabolism^21,22^. Some studies have found that m^6^A methyltransferase-like 3 (METTL3) is involved in growth hormone secretion, cell development, sex hormone synthesis and gonadotropin signalling^23,24^. ALKB homologue 5 (ALKBH5) is highly expressed in the testes, and testis atrophy and fertility are decreased in ALKBH5-knockout mice^22^. However, these symptoms were mild compared with METTL3-knockout mice^25^. YT521 B homology (YTH) domain deficiency, which produces proteins such as YTHDF2 and YTHDC2, decreases the fertility of mice^26,27^. Genetic studies have identified two variants in the fat mass and obesity-associated (*FTO*) gene in three families with delayed puberty^28^. These studies have indicated that m^6^A methyltransferases and demethylases play important roles in maintaining reproductive function and suggest that the overall balance of RNA m^6^A modification plays a critical role in sexual development. Interestingly, FTO is a well-characterised RNA demethylase that modulates appetite and energy metabolism in the ARC of the hypothalamus^29^. More importantly, Yang et al. found that FTO-mediated m^6^A is one of the important factors causing decreases in gamma-amino butyric acid A receptor (GABAAR) levels in GnRH neurons through manganese (Mn)-induced precocious puberty in female rats^30^. However, the biological functions and exact molecular mechanism of FTO-mediated m^6^A modification in puberty onset remain to be elucidated.

Here, we found that FTO in the hypothalamic ARC regulated the onset of puberty and development through RNA demethylase activity. The abundance of m^6^A methylation in the hypothalamus decreased gradually with puberty, while FTO expression increased at the same time in normal female rats at different developmental stages. In addition, RNA m^6^A modification in the hypothalamus was decreased significantly in rats with precocious puberty, while FTO expression was elevated in the precocious puberty rats. In the present study, we sought to determine the biological function of FTO-mediated m^6^A demethylation in the pathogenesis of CPP and investigate the underlying molecular mechanism during puberty onset through the identification of critical mRNA targets of FTO. This study further reveals the pathogenesis of precocious puberty from the perspective of epigenetics.

## Results

### Hypothalamic m^6^A methylation decreased, while FTO increased gradually, with pubertal development

**Fig. 1:**
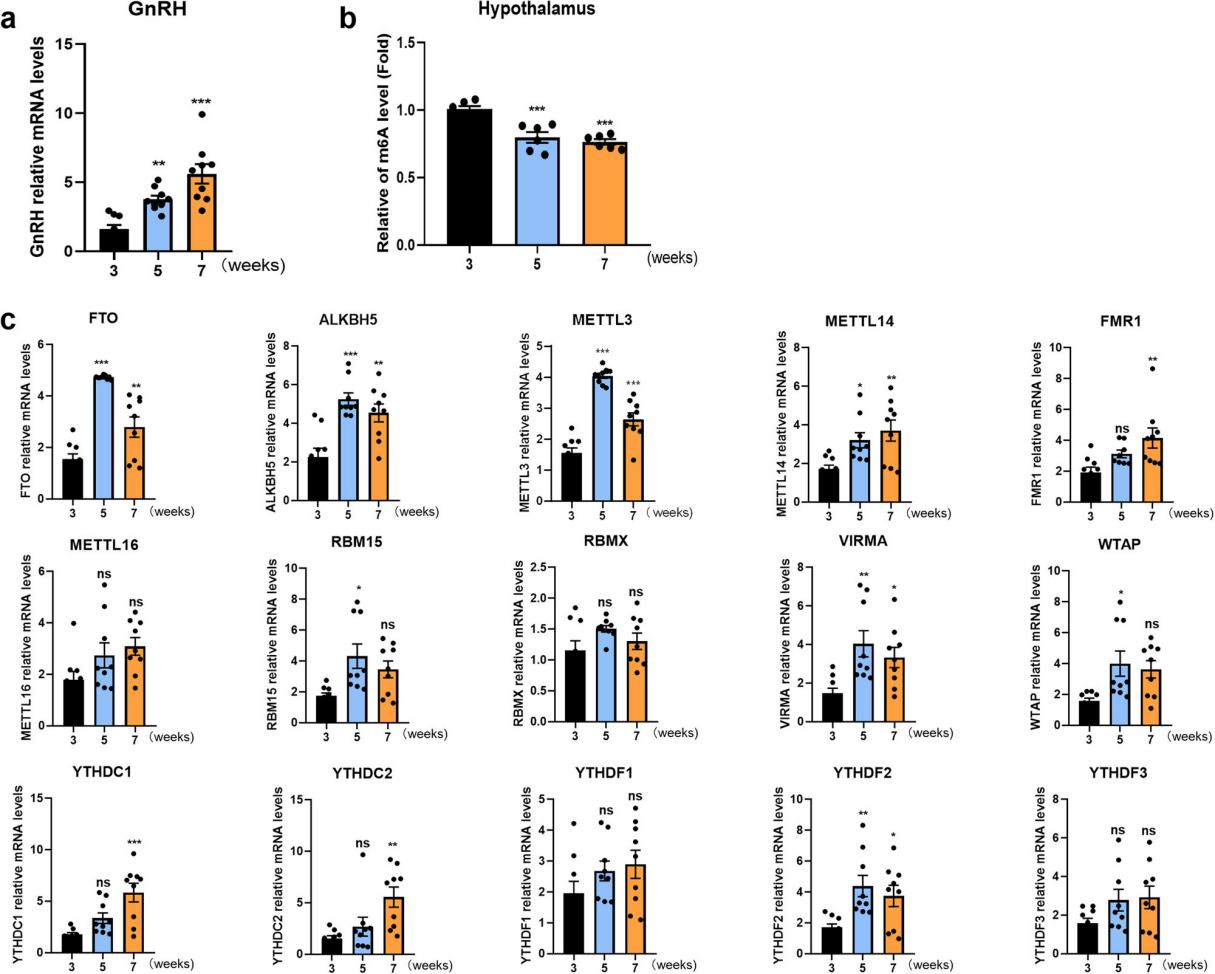
m^6^A methylation decreased, while FTO expression increased, gradually with pubertal development in the female rat hypothalamus. **a** Expression of the *GnRH* gene in the hypothalami of female rats as determined by qPCR (*n* = 9). **b** Colorimetric quantification of m^6^A levels in the hypothalamus at different stages (*n* = 6). **c** mRNA expression of m^6^A-modifying enzymes in the hypothalami of female rats as determined by qPCR (*n* = 9). The bars represent the means ± SEMs. *P < 0.05, **P < 0.01, ***P < 0.001, and ns, *P* > 0.05 versus the 3-week group by one-way ANOVA.

To investigate the function of m^6^A modification in puberty onset, we specifically collected the hypothalami of control female rats at different developmental time points (3 weeks, 5 weeks, and 7 weeks). With the initiation of puberty, the mRNA expression of *GnRH* in the hypothalamus increased significantly, as previously reported^31^ (Fig. 1a). We observed m^6^A peaks that decreased from 3 to 5 weeks using colorimetric analysis (Fig. 1b). These results suggested that m^6^A methylation was decreased from the juvenile stage into early puberty. Key genes that mediate the regulation of RNA methylation m^6^A modification include writers (*METTL3/4/16*, *RBM15*, *WTAP*, *VIRMA*), readers (*YTHDF1/2/3*, *YTHDC1/2*, *RBMX*, *FMR1*) and erasers (*FTO* and *ALKBH5*). Then, quantitative real-time PCR (qRT‒PCR) was used to screen differentially expressed m^6^A methylases in the hypothalami of female rats at different pubertal developmental stages. With regard to writers, the expression of *METTL3* and *VIRMA* first increased and then decreased, reaching a peak at 5 weeks. *METTL14*, *METTL16*, *RBM15* and *WTAP* expression increased gradually with pubertal development. Among readers, *FMR1*, *YTHDC1/2*, and *YTHDF2* showed an increasing trend in expression, while *RBMX* and *YTHDF1/3* showed no significant differences. Among erasers, *FTO* and *ALKBH5* also showed a trend in expression of first increasing and then decreasing, peaking at 5 weeks (Fig. 1c). Previous studies have indicated that single-nucleotide polymorphisms (SNPs) and harmful variations in *FTO* are related to delayed puberty. The above results showed that the changes in *FTO* and m^6^A in the hypothalami of female rats were consistent. m^6^A methylation decreased from 3 to 5 weeks, while *FTO* expression increased in the hypothalami of female rats at the same time.

### Hypothalamic m^6^A methylation decreased, while FTO expression increased, in precocious rats

**Fig. 2:**
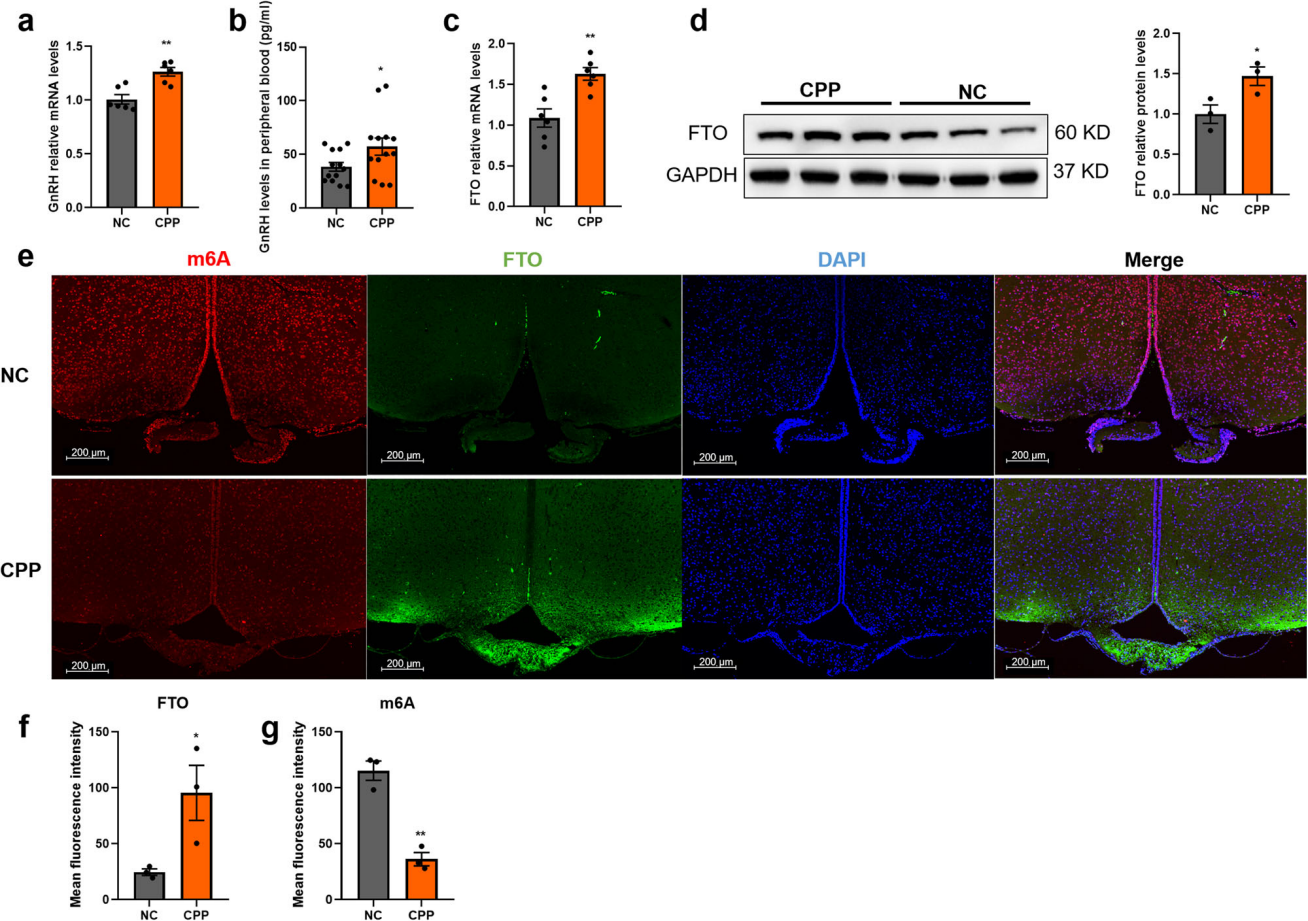
m^6^A methylation was decreased, while FTO expression was increased, in precocious puberty rat models at 3 weeks. **a** *GnRH* mRNA levels detected by qPCR in the hypothalami of 3-week-old female rats (NC, CPP; *n* = 6). **b** GnRH abundance in serum from 3-week-old female rats as determined by ELISA (NC, CPP; *n* = 13). **c** Expression of *FTO* mRNA in the hypothalami of 3-week-old female rats as determined by qPCR (NC, CPP; *n* = 6). **d** Expression of hypothalamic FTO protein in the CPP model as determined by western blotting (NC, CPP; *n* = 3). **e** Abundance of m^6^A (red colour), FTO (green colour) and DAPI (blue colour) in the ARC as determined by IF (NC, CPP; *n* = 3). Scale bars, 200 μm. **f**, **g** Mean fluorescence intensity of FTO and m^6^A as calculated by ImageJ software. The bars represent the means ± SEMs. *P < 0.05, **P < 0.01, and ns, *P* > 0.05 versus the NC group by Student’s *t* test.

To confirm the effect of FTO-mediated m^6^A modification on puberty initiation, CPP female rat models were constructed with danazol. We observed the vaginal opening time at 3 and 5 weeks in these female rats. Nearly half of the females in the CPP group but not in the negative control (NC) group had a vaginal opening at 3 weeks (juvenile stage). The vagina was open in both groups of 5-week-old female rats. Therefore, we chose 3 weeks as the time point for the follow-up experiment (Supplementary Fig. 1a, b). There was no significant difference in body weight between the groups at 3 weeks of age (Supplementary Fig. 1c). *GnRH* mRNA abundance increased significantly at juvenile stages in CPP rats (Fig. 2a). We also compared the expression of GnRH in the serum of female rats at 3 weeks. ELISA revealed that serum GnRH expression in the CPP group increased at 3 weeks (Fig. 2b). We also examined the abundance of LH and FSH in serum using ELISA kits. Although neither of them significantly differed in different groups (Supplementary Fig. 1d, e), LH showed an increasing trend in the CPP groups. We hypothesised that the reason for this phenomenon was that there might be individual differences. Haematoxylin and eosin (H&E) staining showed that there were larger ovaries and more mature follicles in the CPP group than in the NC group (Supplementary Fig. 1f, g). The expression of FTO increased significantly in the hypothalamus in 3-week CPP rats, as shown by qRT‒PCR and western blotting (Fig. 2c, d). Subsequently, 3-week CPP rat brain sections were obtained. The levels and location of m^6^A methylation and FTO were verified by immunofluorescence (IF). m^6^A methylation in the ARC was decreased significantly, while FTO expression was increased in 3-week CPP rats (Fig. 2e–g). These data indicated that FTO-mediated m^6^A demethylation was disrupted with the early onset of puberty and that the m^6^A peak decline was likely to be accelerated in CPP.

### Hypothalamic m^6^A epitranscriptome sequencing in precocious female rats

**Fig. 3:**
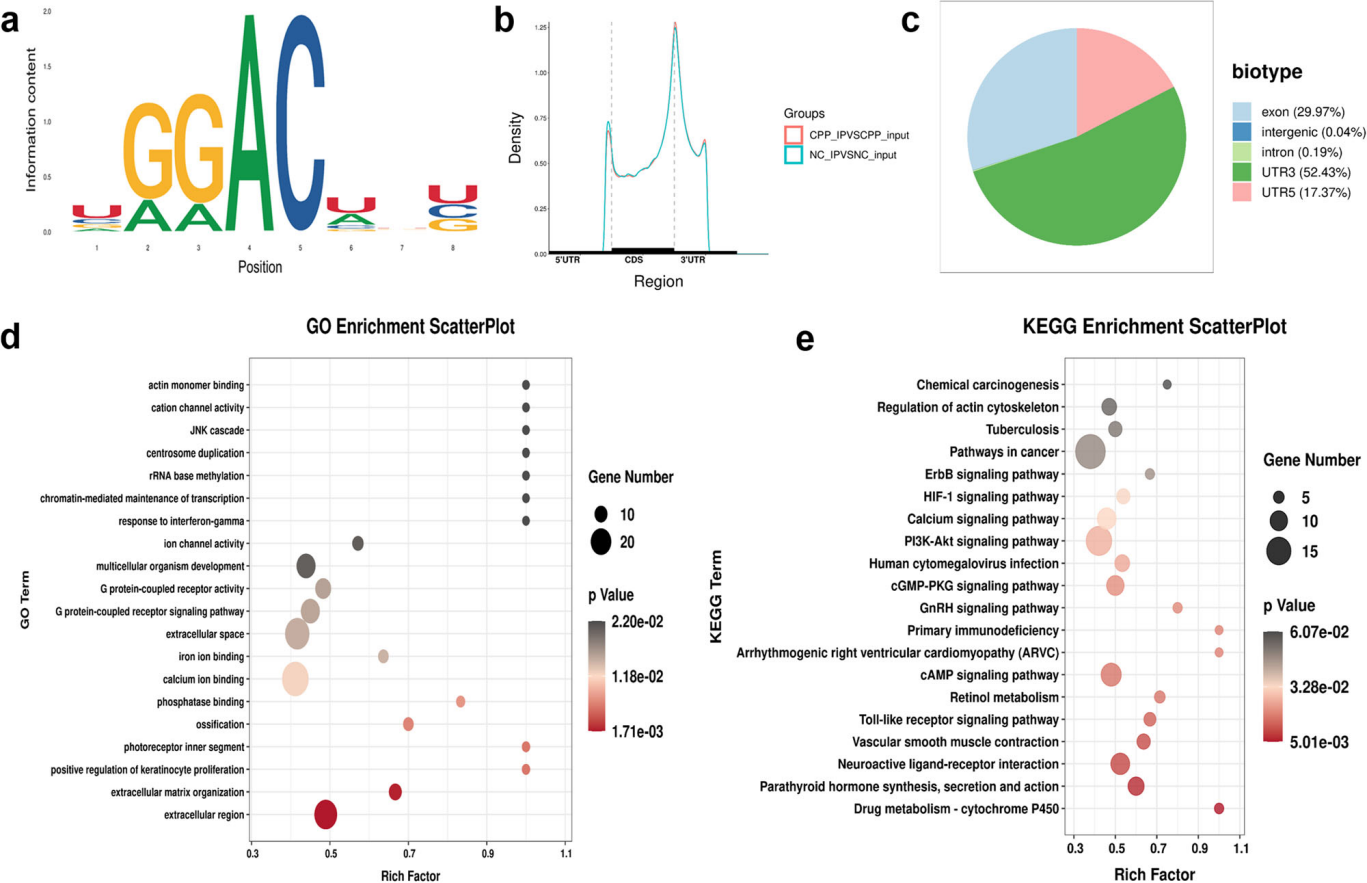
Hypothalamic m^6^A epitranscriptome sequencing in CPP rats at 3 weeks. **a** Most abundant motifs detected in peaks and enriched at peak summits. **b** Distribution of m^6^A peaks throughout the whole mRNA transcript. **c** Proportion of m^6^A peak distribution in exons, intergenic regions, introns, 3′UTRs or 5′UTRs across the entire set of mRNA transcripts. **d** ClusterProfiler identified the enriched GO processes of 613 genes, which showed 1.5-fold m^6^A expression in CPP rat hypothalami compared with NC rat hypothalami. GO analysis of genes with hypomethylated peaks in CPP rats. **e** KEGG pathway analysis of differentially methylated genes in different groups. (NC, CPP; *n* = 3).

The above data demonstrated the expression of FTO across different developmental stages, indicating the importance of m^6^A modification during puberty. Next, we focused on investigating the potential mechanism by which hypothalamic FTO mediates the early onset of puberty. We performed m^6^A epitranscriptome analysis of rat total hypothalamus mRNAs by MeRIP-seq in the CPP and NC groups. In total, 12,649 and 12,703 m^6^A peaks were identified in the NC and CPP rats, respectively. Then, we analysed the differential peaks between the two groups. However, 2334 peaks overlapped in both groups. We also performed an enriched motif search on the detected m^6^A sites and found them to be enriched in the consensus ‘GGAC’ m^6^A motif that was reported previously^32,33^ (Fig. 3a). Consistent with the findings of previous studies^32,34,35^, the majority of m^6^A peaks were preferentially in the 3’ untranslated region (UTR). Next, we analysed the differentially methylated peaks (DMPs) among the different groups (Fig. 3b, c). Similar patterns of the total m^6^A distribution in NC and CPP rats were observed (Supplementary Fig. 2a, b), showing that m^6^A peaks were enriched mainly in the 3’UTR. Comparison of the NC to CPP rat hypothalami revealed that a total of 613 genes showed at least a 1.5-fold change in m^6^A level. Gene Ontology (GO) enrichment analysis of these 613 genes indicated that a handful of genes were associated with calcium ion binding, the extracellular space, and the extracellular region in the hypothalamus during the initiation of puberty (Fig. 3d). Kyoto Encyclopedia of Genes and Genomes (KEGG) pathway analysis showed that multiple gene clusters were enriched in pathways in cancer; phosphatidylinositol 3-kinase (PI3K)-Akt, calcium, cGMP-PKG and cAMP pathways; and neuroactive ligand‒receptor interactions (Fig. 3e). Altogether, the m^6^A-seq data revealed that m^6^A modification occurred on a handful of genes related to the calcium signalling pathway during puberty onset.

### Upregulating FTO promoted GnRH expression by affecting intracellular free Ca^2+^ levels

**Fig. 4:**
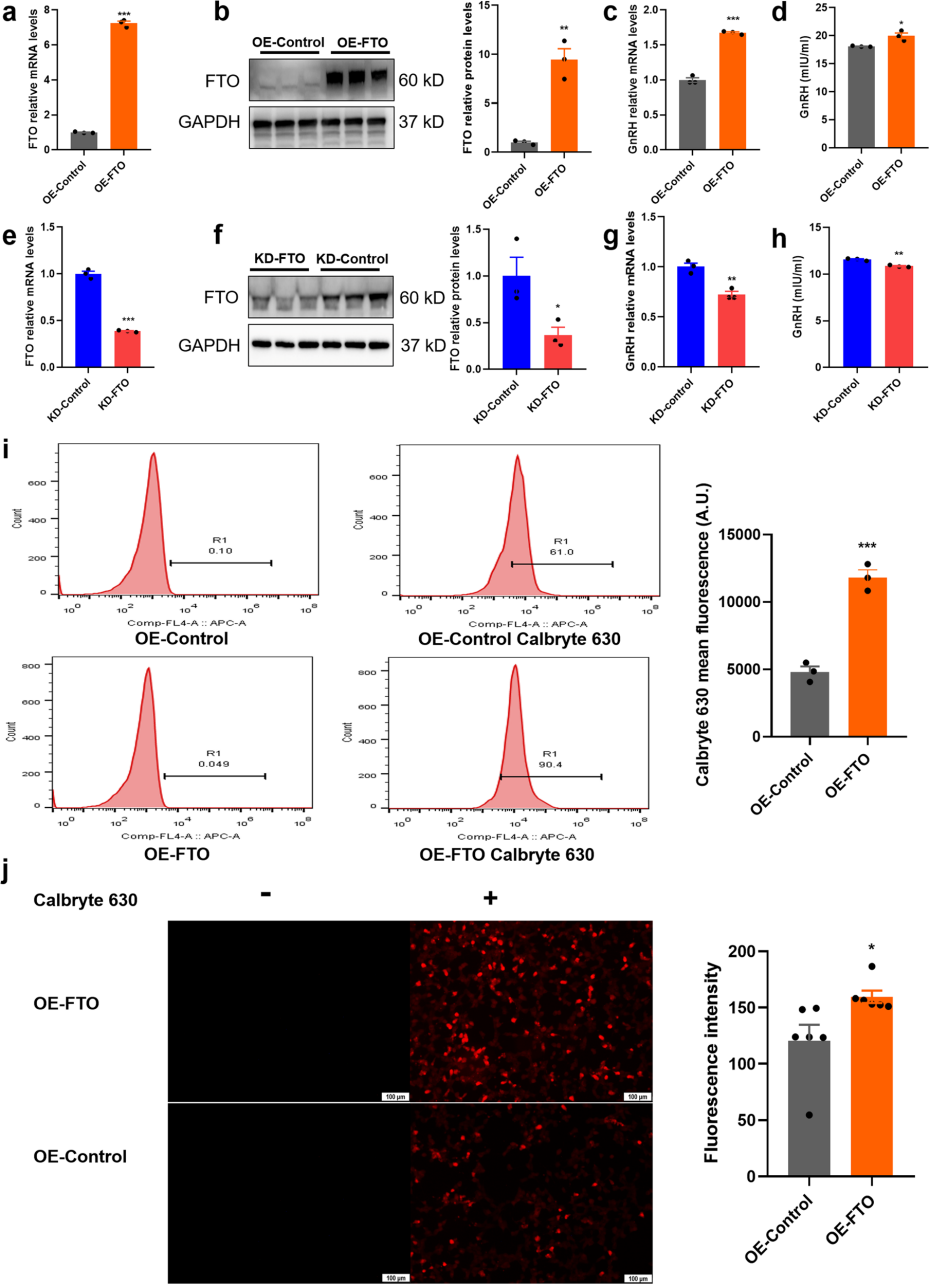
FTO positively regulated GnRH expression by affecting the intracellular levels of free Ca^2+^. **a**, **b** Overexpression of FTO (OE-FTO) in GT1-7 cells as confirmed by qPCR and western blotting (*n* = 3). **c** *GnRH* mRNA levels detected by qPCR in OE-FTO cells (*n* = 3). **d** GnRH abundance in OE-FTO cells as determined by ELISA (*n* = 3). **e**, **f** Knockdown of FTO (KD-FTO) in GT1-7 cells as determined by qPCR and western blotting (*n* = 3). **g** Expression of *GnRH* mRNA as determined by qPCR (*n* = 3). **h** Protein expression of GnRH in KD-FTO cells as determined by ELISA (*n* = 3). **i** Free Ca^2+^ concentrations of OE-Control and OE-FTO GT1-7 cells as determined by fluorescence of Calbryte-630 with flow cytometry as described in the Methods (*n* = 3). Left: flow cytometric histograms of Calbryte-630 and gating with no Calbryte-630 probe as background values. Right: quantitative analysis of cell fluorescence for Calbryte-630. **j** Levels of intracellular free Ca^2+^ (red colour) between OF-FTO and OE-Control cells as determined by IF (*n* = 6). The concentration of fluorescence-labelled Calbryte 630 was 5 μM. Scale bars, 100 μm. The bars represent the means ± SEMs. *P < 0.05, **P < 0.01, ***P < 0.001, and ns, *P* > 0.05 versus OE-control or KD-control group by Student’s *t* test.

To further explore the relationship between FTO and GnRH, we constructed GT1-7 cells with stable knockdown and overexpression of FTO by lentiviral transfection. Efficient overexpression of FTO in cells was validated by qRT‒PCR and western blotting (Fig. 4a, b). Similarly, cells that stably expressed low levels of FTO were successfully constructed (Fig. 4e, f). Overexpression of FTO dramatically promoted the expression of GnRH (a puberty onset marker; Fig. 4c, d). In contrast, FTO knockdown significantly decreased GnRH levels, suggesting that FTO positively regulated GnRH expression (Fig. 4g, h). Previous studies have reported that the transient Ca^2+^ concentrations in GnRH neurons are key factors modulating the expression and pulsatile secretion of GnRH and that a sharp increase in Ca^2+^ abundance can promote pulsatile GnRH release^36^. Subsequently, we found that overexpressing FTO resulted in an increased intracellular free Ca^2+^ concentration by flow cytometry (Fig. 4i). Moreover, IF showed that upregulation of FTO significantly increased intracellular free Ca^2+^ levels (Fig. 4j). Free Ca^2+^ in the cytoplasm regulated GnRH expression through the Ca^2+^ signalling pathway. These results indicated that changes in FTO expression modulated the expression of GnRH by affecting the intracellular levels of free Ca^2+^.

### FTO positively regulated the expression of PLCβ3, affecting the activation of the Ca^2+^/CAMK signalling pathway

**Fig. 5:**
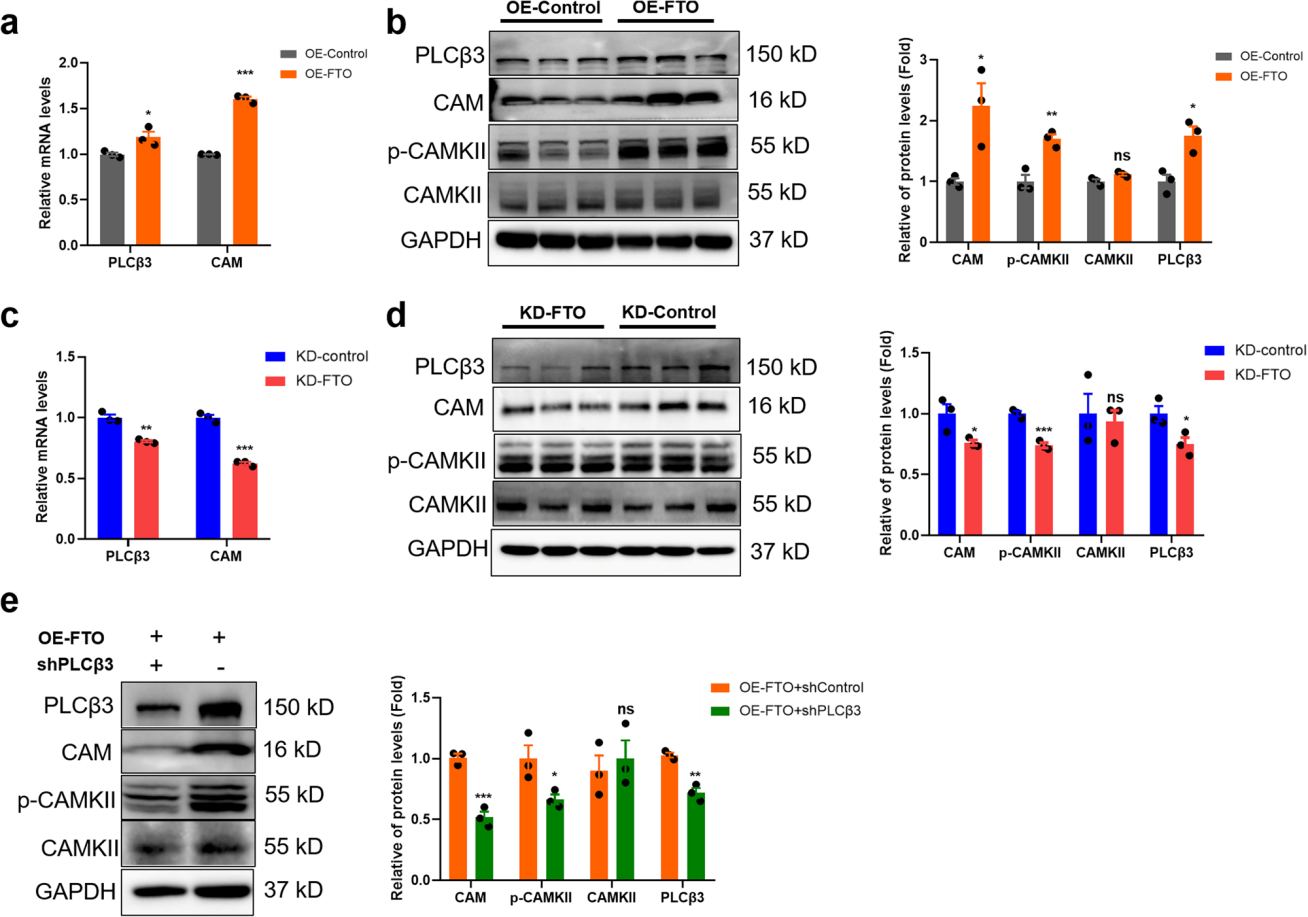
FTO positively regulated PLCβ3 expression, affecting the activation of the Ca^2+^/CAMK signalling pathway. **a** *PLCβ3* and *CAM* (calcium signalling pathway-related) mRNA levels detected by qPCR in OE-FTO cells (*n* = 3). **b** PLCβ3, CAM, CAMKII and phosphorylation-CAMKII protein expression detected by western blotting in OE-FTO cells (*n* = 3). **c** Levels of *PLCβ3* and *CAM* mRNA after knockdown of FTO in GT1-7 cells as determined by qPCR (*n* = 3). **d** Levels of PLCβ3, CAM, CAMKII and phosphorylated CAMKII proteins after knockdown of FTO in GT1-7 cells as determined by western blotting (*n* = 3). **e** Expression of PLCβ3, CAM, CAMKII and phosphorylated CAMKII proteins after knockdown of PLCβ3 in OE-FTO cells as determined by western blotting (*n* = 3). The bars represent the means ± SEMs. *P < 0.05, **P < 0.01, ***P < 0.001, and ns, *P* > 0.05 versus OE-control or KD-control group by Student’s *t* test.

To further investigate potential targets regulated by FTO, we filtered the previous results with GO and KEGG analysis and MeRIP-seq data. We found that the m^6^A methylation of PLCβ3, a key regulator of the Ca^2+^/CAMK pathway, was decreased in CPP rats. Interestingly, our results showed that the mRNA levels of *PLCβ3* and *CAM* were significantly increased following FTO overexpression (Fig. 5a). Consistently, overexpression of FTO markedly elevated PLCβ3, CAM and phosphorylated CAMKII (Fig. 5b) levels. In GT1-7 cells, we also confirmed that downregulating FTO decreased *PLCβ3* and *CAM* gene expression (Fig. 5c). The protein expression levels of PLCβ3 and calcium signalling pathway-related molecules (CAM and p-CAMKII) were also significantly downregulated (Fig. 5d). Next, we cloned PLCβ3 shRNA into FTO-overexpressing GT1-7 cells and found that the effects of FTO overexpression were largely rescued by forced low expression of PLCβ3 (Fig. 5e and Supplementary Fig. 3a, b). These data suggested that PLCβ3 could be the target gene of FTO in GT1-7 cells and that FTO positively regulated GnRH through the PLCβ3/Ca^2+^/CAMK signalling pathway.

### FTO regulated PLCβ3 expression in a targeted, m^6^A-dependent manner

**Fig. 6:**
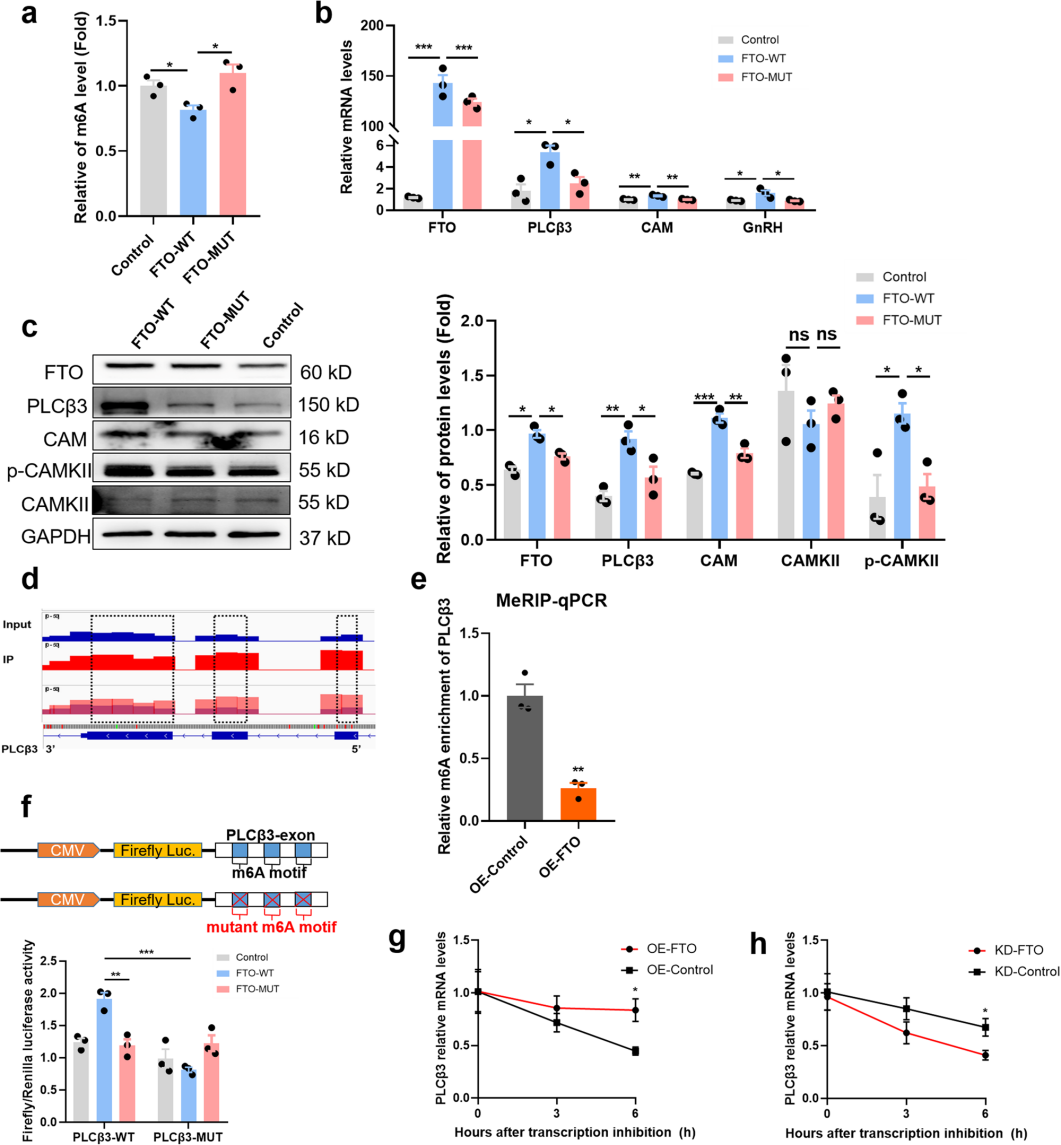
FTO regulated PLCβ3 expression in a targeted, m^6^A-dependent manner. **a** Colorimetric quantification of m^6^A in control, WT-FTO-overexpressing and mutant MUT-FTO-overexpressing GT1-7 cells (*n* = 3). **b** *FTO*, *PLCβ3*, *CAM* and *GnRH* mRNA expression as determined by qPCR in control, WT- and MUT-FTO-overexpressing GT1-7 cells (*n* = 3). **c** FTO, PLCβ3, CAM, CAMKII and phosphorylated CAMKII proteins detected by western blotting in control, WT- and MUT-FTO-overexpressing GT1-7 cells (*n* = 3). The bars represent the means ± SEMs (*n* = 3). *P < 0.05, **P < 0.01, ***P < 0.001, and ns, *P* > 0.05 versus the WT-FTO-overexpressing group by one-way ANOVA. **d** Integrative genomics viewer (IGV) plots of m^6^A peaks at *PLCβ3* mRNA. The y-axis shows the sequence read number, blue boxes represent exons, and blue lines represent introns. **e** MeRIP-qPCR analysis of m^6^A levels of *PLCβ3* mRNA in OE-FTO GT1-7 cells (*n* = 3). The bars represent the means ± SEMs (*n* = 3). **P < 0.01, versus OE-control group by Student’s *t* test. **f** Top panel: schematic diagram of dual-luciferase reporter constructs. Bottom panel: Relative luciferase activity of the WT or MUT (A- to T- mutation) PLCβ3-exon luciferase reporter in control, WT-FTO-overexpressing and MUT-FTO-overexpressing 293 T cells. Firefly luciferase activity was measured and normalised to Renilla luciferase activity. The bars represent the means ± SEMs (*n* = 3). **P < 0.01, ***P < 0.001 versus the WT-FTO-overexpressing group by one-way ANOVA. **g**, **h** *PLCβ3* mRNA stability assay after transfection of GT1-7 cells with FTO overexpression or knockdown lentivirus. The bars represent the means ± SEMs (*n* = 3). *P < 0.05 versus the control group by two-way ANOVA.

To further elucidate the underlying molecular mechanism of FTO in GnRH regulation, we constructed wild-type (FTO-WT) and catalytic mutant FTO^R96Q^ (FTO-MUT) plasmids^37,38^ to determine whether the demethylase activity of FTO was needed for its effect on GnRH. The impact of FTO-WT or FTO-MUT on cellular m^6^A levels was confirmed by colorimetric analysis (Fig. 6a). Next, we investigated whether FTO influenced the expression of PLCβ3 through RNA demethylation. As expected, we found that in GT1-7 cells, overexpression of wild-type FTO, but not mutant FTO, substantially increased the mRNA expression of *PLCβ3*, *CAM*, and *GnRH* (Fig. 6b). Overexpressing FTO-WT, but not FTO-MUT or an empty vector, significantly increased PLCβ3, CAM, and p-CAMKII protein levels (Fig. 6c). According to m^6^A-seq data and motifs predicted by Integrative Genomics Viewer (IGV), the sites with altered m^6^A modification levels were mainly enriched in exonic regions of PLCβ3 (Fig. 6d). However, there were no significant changes in the 5’UTR or 3’UTR. Gene-specific methylated RNA immunoprecipitation-qPCR (MeRIP-qPCR) assays demonstrated that FTO overexpression significantly decreased the m^6^A levels on mRNA transcripts of *PLCβ3* (Fig. 6e). More importantly, to assess whether m^6^A modification of target mRNAs was necessary for FTO-mediated gene regulation, we performed dual-luciferase reporter and mutagenesis assays in GT1-7 cells. Overexpression of FTO-WT, but not FTO-MUT, substantially increased the activity of luciferase containing wild-type exon fragments of PLCβ3 (Fig. 6f). The increase was abrogated when the m^6^A sites were mutated (A was replaced with T; Fig. 6f), demonstrating that FTO regulated the m^6^A demethylation of PLCβ3 by binding to specific motifs. Next, we further investigated whether the changes in m^6^A methylation affected *PLCβ3* mRNA degradation in GT1-7 cells. In the presence of actinomycin D, an inhibitor of mRNA transcription, FTO overexpression delayed the degradation of *PLCβ3* mRNA, whereas FTO knockdown significantly accelerated this process (Fig. 6g, h). These results indicate that m^6^A modification is likely to affect *PLCβ3* mRNA levels, at least partly by regulating mRNA stability. Taken together, our data illustrate that FTO-mediated PLCβ3 regulation relies on the demethylase activity of FTO and on m^6^A modifications in the target motifs.

### ARC-targeted overexpression of FTO promoted early puberty onset through the PLCβ3/Ca^2+^/CAMK signalling pathway

**Fig. 7:**
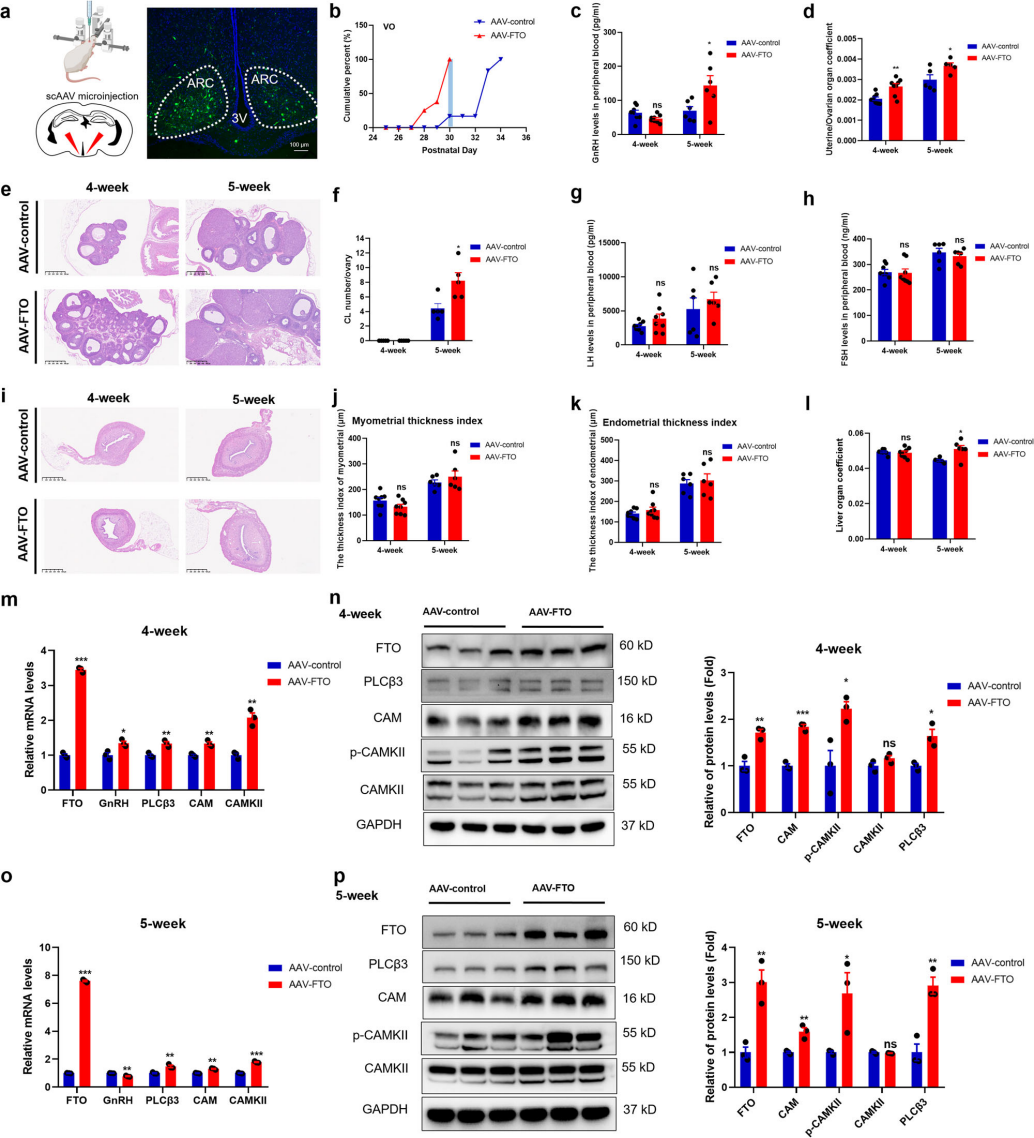
Overexpression of FTO in the ARC promoted early puberty onset through the PLCβ3/Ca^2+^/CAMK signalling pathway. **a** Left panel: schematic illustrations of scAAV microinjection. The picture material of mouse was created with BioRender.com. Right panel: transduction of ARC cells with scAAV delivered to the ARC in female rats. Red triangle: microinjection site. Green: scAAV-infected cells; blue: DAPI-stained cell nuclei in the hypothalamus; dotted line: ARC. 3 V, third ventricle. Scale bars, 100 μm. **b** Cumulative percentages of vaginal opening (VO) after intra-ARC AAV-FTO and AAV-control transfection. Blue shading depicts the time when all AAV-FTO animals showed VO. **c** GnRH abundance in serum at 4 weeks (*n* = 8) and 5 weeks (*n* = 6) in female rats as determined by ELISA. **d** Uterine/ovarian coefficients in the rats transfected with AAV-control or AAV-FTO at different stages (*n* = 5–8). **e**, **f** Pathological assessment of follicular development by H&E staining in the ARC-transfected group (**e**) and the number of corpora lutea (**f**) (*n* = 5). Scale bars, 500 μm. **g**, **h** Serum LH (**g**) and FSH (**h**) levels in 4-week-old (*n* = 8) and 5-week-old (*n* = 6) female rats as determined by ELISA in the ARC-transfected group. **i**–**k** Pathological assessment of the uterus by H&E staining in the ARC-transfected group (**i**) and the myometrial thickness index (**j**) and endometrial thickness index (**k**) between the groups. Scale bars, 500 μm. **l** Liver coefficient in the rats transfected with AAV-control or AAV-FTO at different stages (*n* = 6–8). **m**, **o** *FTO*, *PLCβ3*, *CAM*, and *CAMKII* mRNA levels detected by qPCR in the ARC in 4-week (**m**) or 5-week (**o**) female rats receiving AAV-control (*n* = 3) or AAV-FTO (*n* = 3). **n**, **p** FTO, PLCβ3, CAM, CAMKII and phosphorylated CAMKII proteins detected by western blotting in the ARC in 4-week (**n**) or 5-week (**p**) female rats receiving AAV-control (*n* = 3) or AAV-FTO (*n* = 3). Uterine/ovarian organ coefficient = [uterine/ovarian weight (g)]/body weight (g). Liver organ coefficient = liver weight (g)]/body weight (g). AAV-FTO for the FTO-overexpression group, AAV-control for the AAV negative control group. The bars represent the means ± SEMs (*n* = 3). *P < 0.05, **P < 0.01, ***P < 0.001, and ns, *P* > 0.05 versus the AAV-control group by Student’s *t* test.

To determine whether overexpression of FTO affects the timing of puberty and to elucidate its related mechanisms, we employed a self-complementary adeno-associated virus (scAAV) as a transgenic tool to specifically overexpress FTO. Unlike single-stranded AAV (ssAAV), scAAV does not need to undergo a single-stranded-to-double-stranded conversion process and can be directly expressed after entering the cell. The scAAV was expressed 3 days postinjection, and its effect was sustained for at least 3 months. We delivered scAAV constructs to the ARC in 3-week-old female rats via stereotactic injection according to the Rat Brain Atlas (Paxinos and Watson, Fifth Edition) and previous position information^39^. Control animals were injected with a scAAV construct carrying an enhanced green fluorescent protein (GFP). We observed that the scAAV reached the ARC 1 week postinjection (Fig. 7a). The rats were observed daily for three days after the operation. Hypothalamic tissues and serum were collected at 4 weeks and 5 weeks. We found that body weight was not different between the AAV-FTO and AAV-control groups (Supplementary Fig. 4a). Female rats in the AAV-FTO group had early vaginal opening (Fig. 7b). Rats injected with AAV-FTO showed an obviously increased GnRH abundance in serum at 5 weeks but no alteration at 4 weeks (Fig. 7c). Moreover, there was a significant increase in the uterine/ovarian organ coefficient in the AAV-FTO group at 4 weeks and 5 weeks (Fig. 7d). These results demonstrated that FTO regulated puberty onset and reproductive function. We used H&E staining to observe uterine and ovarian morphology and function. Compared with AAV-control rats, AAV-FTO rats showed enlarged ovaries (Fig. 7e). At 5 weeks, the corpus luteum (CL) numbers were increased significantly in the ARC-specific AAV-FTO group (Fig. 7f). Subsequently, we examined the abundance of LH and FSH in serum using ELISA kits. Although neither of them significantly differed, LH showed an increasing trend in both phases (Fig. 7g, h). The H&E staining results showed no obvious change in the uterus (Fig. 7i), and there were no significant differences in the myometrial thickness index and endometrial thickness index (Fig. 7j, k). The liver organ coefficient appeared to obviously increase in the early puberty stage (5 weeks), although there was no significant difference at 4 weeks (Fig. 7l). Similarly, fat H&E staining showed that the white fat of the rats in the AAV-FTO group had large vacuoles and gradually fused with disorganised tissues (Supplementary Fig. 4b). To further determine whether the overexpression of FTO in the ARC resulted in changes in the PLCβ3 and Ca^2+^/CAMK signalling pathways, we collected hypothalami at 4 weeks and 5 weeks for mRNA and protein quantitation. The expression of FTO was increased significantly in the ARC AAV-FTO groups. In agreement with the in vitro results, animals in the AAV-FTO groups showed pronounced increases in *PLCβ3*, *CAM* and *CAMKII* mRNA abundance at 4 weeks and 5 weeks (Fig. 7m, o). Consistent with the in vitro data, FTO overexpression (AAV-FTO) increased PLCβ3 expression and activated the Ca^2+^ signalling pathway at 4 weeks (Figs. 7n) and 5 weeks (Fig. 7p). To further confirm that PLCβ3 was the direct target of FTO, the brains of female rats in the AAV-FTO group and the control group were subjected to immunofluorescence staining for PLCβ3. The results showed that PLCβ3 increased significantly when FTO was overexpressed in the ARC of the hypothalamus (Supplementary Fig. 4c, d). Previous studies have shown that Kiss1 plays an important role in regulating GnRH during puberty initiation. However, we found that *Kiss1* mRNA decreased significantly when FTO increased in the ARC at 4 weeks and 5 weeks (Supplementary Fig. 4e, f). Thus, prepubertal overexpression of FTO in the ARC promoted early puberty onset in female rats by increasing the expression of PLCβ3 and activating the Ca^2+^ signalling pathway.

## Discussion

**Fig. 8:**
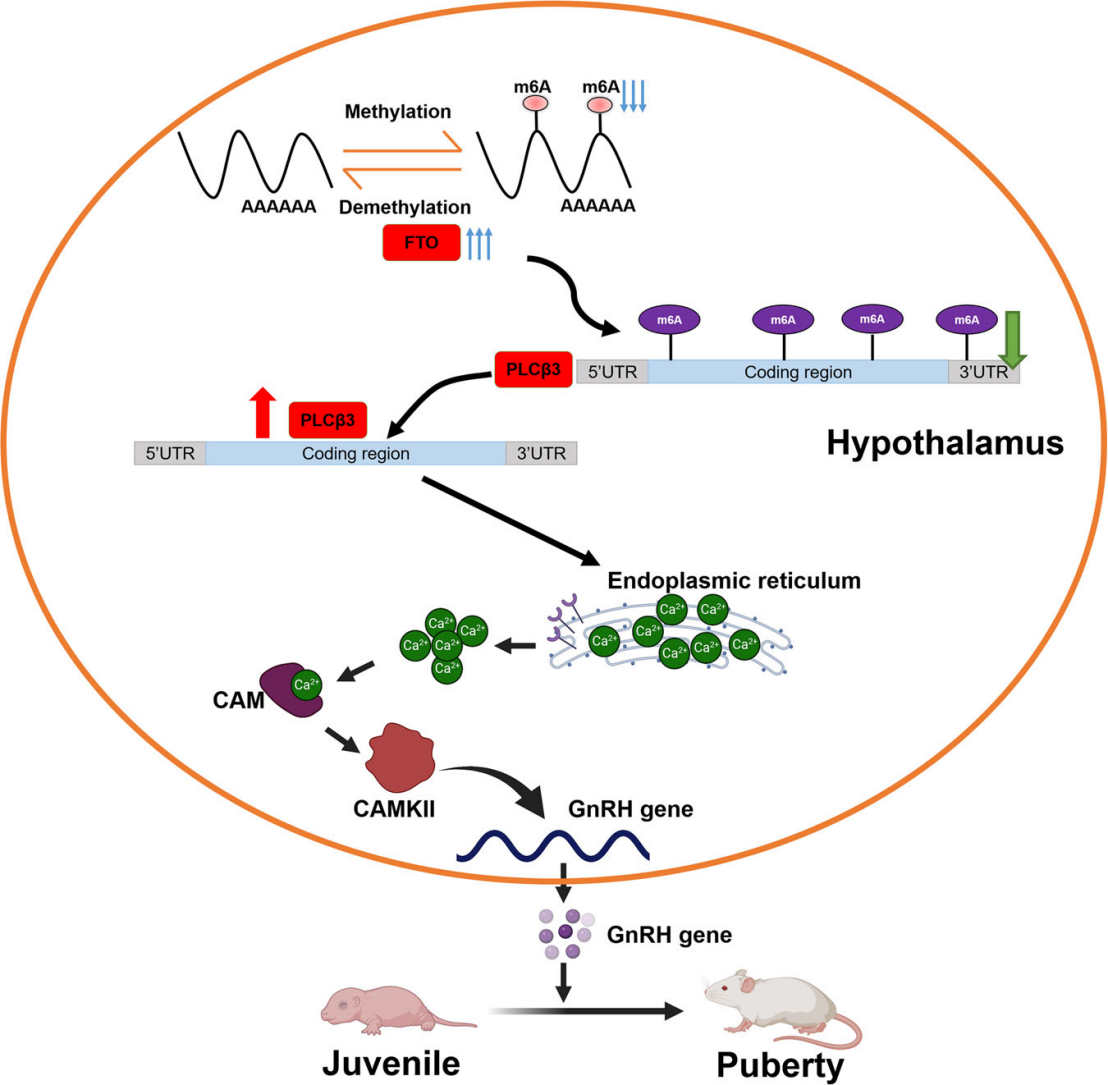
Schematic illustration summarising the findings of the current work. FTO expression increases gradually with puberty onset and peaks at the puberty stage. Overexpression of FTO leads to decreased m^6^A modification levels of *PLCβ3* mRNA, which results in higher mRNA and protein levels of PLCβ3. The enhancement of PLCβ3 can activate the Ca^2+^ signalling pathway to promote GnRH expression. Overexpression of FTO in the ARC of the hypothalamus also promotes early puberty and disrupts ovarian development. The picture materials were downloaded from BioRender (https://www.biorender.com).

In the current study, the expression of hypothalamic m^6^A was decreased in adolescent and CPP female rats. The expression of FTO showed the opposite trend. MeRIP-seq identified PLCβ3 as one of the targets modified by FTO. Upregulating FTO promoted PLCβ3 expression by reducing m^6^A methylation on *PLCβ3* mRNA, thus promoting GnRH expression. In addition, FTO overexpression increased intracellular free Ca^2+^ abundance and activated the Ca^2+^ signalling pathway. Forced low expression of PLCβ3 reversed this phenomenon. Furthermore, prepubertal-specific upregulation of FTO in the ARC resulted in early vaginal opening and ovarian development by promoting the expression of PLCβ3 and activating the Ca^2+^ signalling pathway. This led to increased expression of GnRH and promoted pubertal development (Fig. 8). m^6^A expression showed a decreasing trend during pubertal development. This change was consistent with the findings of a previous study in mice^40^. However, we only explored the changes in m^6^A and FTO at 3 weeks; we did not further study the variations in m^6^A levels and FTO expression at puberty in CPP rats. The main reason was that the female rats in both groups all had a vaginal opening at 5 weeks old and began to enter the stage of pubertal development. Compared with the trend of m^6^A in the hypothalamus in control pubertal female rats, we found that m^6^A levels decreased in advance during precocious puberty, which in turn caused early pubertal development. Previous studies have shown that harmful mutations in *FTO* cause delayed puberty^28^. However, the role of FTO in the pathophysiology of puberty remains largely unknown. The present study demonstrated that the peak FTO expression appeared early in CPP rats. In conclusion, we explored the epigenetic mechanism of FTO-mediated m^6^A methylation on puberty initiation and found that this methylation plays a critical epigenetic role in the initiation of puberty.

GO and KEGG analysis of hypothalamic m^6^A epitranscriptome sequencing in precocious female rats showed that most of the differentially expressed genes affecting puberty development were enriched in calcium signalling pathways. Previous studies have shown a correlation between the Ca^2+^ signalling pathway and GnRH expression. We analysed related genes enriched in the calcium ion signalling pathway and found that the m^6^A modification of *PLCβ3*, *calm* and *camk* located in this pathway all changed. Therefore, we focused our attention on *PLCβ3*, which is upstream of the Ca^2+^ signalling pathway. MeRIP co-RNA sequencing assays and functional studies confirmed that *PLCβ3* is a crucial target gene downstream of FTO. Phospholipase Cβ (PLCβ) is located in the endoplasmic reticulum (ER) and regulates the release of Ca^2+^, which regulates CAM/CAMK protein expression to modulate downstream gene expression. Moreover, intracellular free Ca^2+^ can induce action potentials in nerve cells and promote the secretion of neurotransmitters^41,42^. In this research, we unexpectedly found that changes in FTO expression affected intracellular free Ca^2+^ levels and activated the Ca^2+^ signalling pathway. Overexpression of FTO increased the concentration of free Ca^2+^ in the cytoplasm, which promoted GnRH expression. The overexpression of FTO reduced the m^6^A level of PLCβ3 mainly in exons, which in turn led to upregulation at the mRNA level and especially at the protein level. Surprisingly, we found that over 5% of potential FTO targets tended to be positively regulated by FTO. In addition, we found that a handful of genes were enriched in the PI3K/Akt signalling pathway. By analysing the differentially expressed genes enriched in this pathway, we identified some potential targets of FTO, such as BDNF and GABAR. We verified these results in further experiments. Previous studies have suggested that mRNA transcripts with m^6^A modifications tend to be less stable^43^. As shown in Fig. 6g, h, FTO overexpression alleviated the degradation of *PLCβ3* mRNA, and knockdown of FTO accelerated the degradation of *PLCβ3* mRNA. Our data further suggest that an additional reading process may exist that controls the stability of FTO target transcripts. It will be very interesting to uncover such an alternative reading process in the future. Moreover, FTO is known to possess demethylase activity for m^6^A in mRNAs. Both previous works and our present study show that m^6^A is not evenly distributed across transcripts but rather specifically enriched around stop codons, in 3’UTRs, and within long internal exons of RNA^35,44,45^. Alternatively, the modulation of PLCβ3 expression by FTO may rely on demethylase activity to a certain extent.

While m^6^A is highly expressed in the entire hypothalamus, FTO is highly expressed within the ARC (shown in Fig. 2e). Most importantly, the ARC nucleus in the hypothalamus is one of the important positions controlling reproductive development in female rats. Therefore, we specifically overexpressed FTO in the ARC nucleus by stereotaxic injection. Consistent with our above results, we found that FTO overexpression in the ARC of female rats achieved by stereotaxic injection in juveniles increased PLCβ3 expression and activated the Ca^2+^ signalling pathway. The onset of puberty requires a corresponding energy supply, and increased fat mass is associated with the early initiation of puberty^46^. Epidemiological studies have demonstrated a strong association between *FTO* SNPs or overweight/obesity and the risk of various types of cancers, such as breast, prostate, kidney, and pancreatic cancers^47,48^. Therefore, it is possible that increased expression of FTO caused by obesity-associated SNPs^49,50^ may contribute (to some extent) to the increased risk of individuals with overweight and obesity developing various types of disorders of sex development. However, there were no differences in body weight between the AAV-FTO and AAV-control groups. FTO-deficient mice have been found to show significant weight loss^51^. This phenomenon is inconsistent with our study. The main reason for this inconsistency was that we only intervened in FTO expression within the ARC. However, FTO is expressed in various tissues, and manipulation of FTO only in the ARC is insufficient to cause weight gain in rats. In addition, the female rats showed early vaginal opening and ovarian and uterine development in the AAV-FTO groups. Another interesting phenomenon is that the levels of GnRH increased at 4 weeks in the AAV-FTO-infected groups but decreased at 5 weeks. We suspected that GnRH was a kind of reproductive hormone synthesised and secreted by the hypothalamus. Its expression includes two parts: synthesis and release. We detected peripheral blood serum by ELISA and found that GnRH secretion increased significantly in the AAV-FTO group at 5 weeks. This cumulative effect might be one of the reasons for the decrease in *GnRH* mRNA synthesis at this time. At the same time, we found that *Kiss1* mRNA decreased significantly when FTO increased. Kiss1 neurons have attracted particular attention for their role as a key factor in the brain’s control of the onset of puberty. However, whether kisspeptins are a true trigger of puberty or whether they act as an amplifier of cascades of events leading to the full activation of GnRH neurons at the onset of puberty remains controversial. According to a recent study, some maternal age at menarche (AAM)-related genetic variants were associated with birth weight (BW), including genes such as *FTO* and *Kiss1*^52^. However, the regulatory relationship between *FTO* and *Kiss1* is not yet known. It would be interesting to further investigate the specific regulatory mechanisms of *FTO* and *Kiss1* in the future. Thus, prepubertal overexpression of FTO in the ARC selectively resulted in early vaginal opening and ovarian development by promoting PLCβ3 expression and activating the Ca^2+^ signalling pathway. The limitation of the study is that only female rats and GT1-7 cells were employed. Further work investigating the roles of FTO and m^6^A in patients with CPP may be helpful for further elucidating the potential mechanism and targets underlying disorders of sex development.

In summary, we provide compelling in vitro and in vivo evidence demonstrating that alterations in FTO-catalysed m^6^A modification contribute to the onset of puberty by modulating the Ca^2+^ pathway. Our study highlights the functional importance of the m^6^A modification machinery in CPP and provides a novel epigenetic regulatory mechanism. Recently developed specific FTO inhibitors are promising for the treatment of obesity^53^. In addition, given the functional importance of FTO in CPP, targeting the FTO/PLCβ3/Ca^2+^/CAMK axis is a promising and attractive therapeutic approach for CPP. This work will aid in the discovery of new therapeutic targets and improve the prognosis of the disease.

## Methods

### Animal groups and CPP rat model construction

Three-, 5-, and 7-week-old Sprague‒Dawley female rats and their mothers were obtained from Shanghai Jie Sijie Experimental Animal Co., Ltd. (licence number: #SCXK2018-0004) and housed under a 12 h/12 h light/dark cycle at a controlled temperature. The female rats were randomly divided into the following groups: 3-week, 5-week, 7-week, CPP model, and CPP model with vehicle (as a negative control). To establish CPP models, 5-day-old female rats were administered 300 μg of danazol (Sigma‒Aldrich, Germany) dissolved in 30 μL of vehicle (propylene glycol:ethanol, 1:1, v/v) as previously reported^54^. Danazol significantly accelerated the opening of the vulva, the establishment of two regular sexual cycle times, and uterine wall thickening. The time of uterine and ovarian development was significantly earlier in the danazol group than in the control group. Moreover, the cytological changes in vaginal discharge induced by danazol in rats with precocious puberty were consistent with those in adult rats. The expression of *GnRH* mRNA in the hypothalamus was increased, and the release of GnRH in the hypothalamus was accelerated. These results indicated that danazol accelerated the maturation of the HPGA and that the model of precocious puberty induced by danazol was true precocious puberty. Body weight and vaginal opening were examined daily in the CPP model groups. All female rats were included for testing of the hypothalamus and ovary. We have complied with all relevant ethical regulations for animal testing. This animal experiment was authorised by the Animal Experiment Committee of Shanghai Children’s Hospital, School of Medicine, Shanghai Jiao Tong University (SHCH-IACUC-2022-XMSB-40).

### Quantification of m^6^A modification

A SteadyPure Universal RNA Extraction Kit (AG, China) was used for RNA extraction. A Dynabeads mRNA purification kit (Invitrogen, USA) was utilised for polyadenylated mRNA purification according to the manufacturer’s instructions. We used an EpiQuik m^6^A RNA Methylation Quantification Kit (Colorimetric) (Epigentek, USA) to measure the total RNA m^6^A level. Two hundred nanograms of RNA was injected into each assay well, and the capture antibody with a suitably diluted solution concentration was added and mixed. The m^6^A content was detected by colorimetry at a wavelength of 450 nm and calculated according to the standard curve.

### MeRIP-seq

Total cellular RNA was purified from fresh female rat hypothalami using TRIzol Reagent (Invitrogen, Carlsbad, CA, USA) according to the manufacturer’s protocol. The RNA amount and purity of each sample were quantified using a NanoDrop (Thermo Fisher Scientific, Waltham, MA, USA). The RNA integrity was assessed with a Bioanalyzer 2100 (Agilent, CA, USA) with RIN > 7.0 and confirmed by electrophoresis with denaturing agarose gel. Approximately 25 μg of total RNA representing a specific adipose type was used to deplete ribosomal RNA according to the Epicentre Ribo-Zero Gold Kit (Illumina, San Diego, USA). Following purification, the ribosomal-depleted RNA was fragmented into small pieces using a Magnesium RNA Fragmentation Module (NEB, USA) at 86 °C for 7 min. Then, the cleaved RNA fragments were incubated for 2 h at 4 °C with m^6^A-specific antibody (Synaptic Systems, Germany) in IP buffer (50 mM Tris-HCl, 750 mM NaCl and 0.5% Igepal CA-630). Then, the IP RNA was reverse-transcribed to generate cDNA with SuperScript II Reverse Transcriptase (Invitrogen, USA), which was next used to synthesise U-labelled second-stranded DNA with *E. coli* DNA polymerase I (NEB, USA), RNase H (NEB, USA) and dUTP Solution (Thermo Fisher, USA). An A-base was then added to the blunt end of each strand, preparing the strands for ligation to the indexed adapters. Each adapter contained a T-base overhang for ligating the adapter to the A-tailed fragmented DNA. Single- or dual-index adapters were ligated to the fragments, and size selection was performed with AMPureXP beads. After heat-labile UDG enzyme (NEB, USA) treatment of the U-labelled second-stranded DNAs, the ligated products were amplified by PCR under the following conditions: initial denaturation at 95 °C for 3 min; 8 cycles of denaturation at 98 °C for 15 s, annealing at 60 °C for 15 s, and extension at 72 °C for 30 s; and then final extension at 72 °C for 5 min. The average insert size for the final cDNA library was 300 ± 50 bp. Finally, we performed 2 × 150 bp paired-end sequencing (PE150) on an Illumina NovaSeq 6000 (LC-Bio Technology Co., Ltd., Hangzhou, China) following the vendor’s recommended protocol.

### RNA sequencing

Total RNA was extracted from fresh female rat hypothalami using TRIzol Reagent (Invitrogen, USA) for RNA sequencing. Residual genomic DNA was removed, and RNA was purified following standard instructions. All samples used for the cDNA library had excellent purity as assessed by NanoDrop 2000, and the RNA integrity value showed no visible signs of degradation in an Agilent 2100 Bioanalyzer (Agilent Technologies Inc., Santa Clara, CA, USA). The extracted mRNA was fragmented, reverse-transcribed into cDNA, and ligated with proprietary adapters to the 3’ and 5’ termini. Next, paired-end sequencing was performed with Illumina HiSeq sequencing technology (Illumina, San Diego, USA).

### Cell culture

GT1-7 cells are subset strains of GT1 cell lines, which are GnRH neuron cell lines isolated from the hypothalami of transgenic mice and have the typical characteristics of highly differentiated neuroendocrine cells. They are ideal in vitro cell models. The GT1-7 cells used in this study were kindly provided by the Shanghai Clinical Center for Endocrine and Metabolic Diseases, Shanghai Jiao Tong University. 293 T cells were purchased from the Cell Bank of the Chinese Academy of Sciences (Shanghai, China). The cells were routinely tested for mycoplasma contamination. The cells were cultured in Dulbecco’s modified Eagle’s medium (DMEM, Gibco, NY, USA) supplemented with 10% foetal bovine serum (FBS, Gibco), 100 U/mL penicillin and 100 μg/mL streptomycin. The cells were incubated in a 37 °C incubator with 5% CO_2_.

### RNA extraction and qRT‒PCR

Total RNA was extracted from the hypothalamus and cultured cells using TRIzol Reagent (Invitrogen, CA, USA) following the manufacturer’s instructions. One microgram of total RNA was reverse-transcribed into cDNA using a reverse transcription system (AG, Hunan, China). qRT‒PCR was performed using a SYBR Premix Pro Taq HS qPCR Kit (AG, Hunan, China). Relative gene expression was analysed based on the 2^-ΔΔCt^ method with β-actin as an internal control. At least three independent experiments were analysed. All primers (Supplementary Table 1) were synthesised by Shanghai Sangon Biotech Co., Ltd.

### Western blot analysis

The protein component of the cells was lysed with M-PER^®^ Mammalian Protein Extraction Reagent lysis buffer (Thermo, MI, USA) containing protease inhibitor and PhosStop (Roche, Switzerland). After quantification with a BCA protein assay kit (Thermo, MI, USA), total proteins (30 μg) were loaded on a 10% or 6% sodium dodecyl sulfate‒polyacrylamide gel and separated by electrophoresis. Then, the protein was transferred to a 0.22 μm polyvinylidene fluoride (PVDF) membrane (Millipore, MA, USA). After blocking with QuickBlock buffer (Beyotime Biotechnology, Shanghai, China), the membranes were incubated with rabbit anti-FTO (1:1000, ABclonal, Wuhan, China), rabbit anti-CAM (1:1000, ABclonal, Wuhan, China), rabbit anti-CAMKII (pan) (1:1000, Cell Signaling Technology, USA), rabbit anti-phospho-CAMKII (Thr286) (1:1000, Cell Signaling Technology, USA), rabbit anti-PLCβ3 (1:1000, Cell Signaling Technology, USA) and rabbit anti-GAPDH (1:1000, Cell Signaling Technology, USA) overnight at 4 °C. Subsequently, the membranes were rinsed three times with Tris-buffered saline with 0.1% Tween 20 (TBST) and probed with an HRP-labelled secondary antibody (1:5000, ABclonal, Wuhan, China) at room temperature for 1 h. After three additional rinses with TBST, the membranes were visualised using an enhanced chemiluminescence (ECL) system. The greyscale values of the protein bands were analysed using ImageJ software. The primary antibodies used in the experiment are listed in Supplementary Table 2. The uncropped gel images are shown in Supplementary Fig. 5.

### Reporter construction, transfection and scAAV packing and administration

Specific plasmid vectors of *PLCβ3* were designed and synthesised by OBiO Technology (Shanghai, China) to silence *PLCβ3* expression in GT1-7 cells. The *FTO* overexpression and knockdown constructs were based on the lentiviral vector pHIV. qRT–PCR and western blotting were performed to assess the effectiveness of the plasmid vectors, overexpression-FTO (OE-FTO) lentivirus, and knockdown-FTO (KD-FTO) lentivirus. The targeted knockdown vectors with the best silencing effects were utilised for the following experiments. The related sequences of the vectors were as follows: KD-FTO-1: 5′- GCTGAGGCAGTTCTGGTTT -3′; KD-PLCβ3-1: 5′- CGCGGGAGTAAGTTCATCAAA -3′. The specific plasmid vectors were transfected into cells using Lipofectamine 3000 (Invitrogen, USA) according to the manufacturer’s protocol. The GT1-7 cells were collected for the following experiments 48 h or 72 h later. Rat *FTO* cDNA was cloned and inserted into the scAAV expression vector with an EFFS or CMV promoter. The scAAV-FTO and scAAV-control were packaged by Vigene Biosciences (Shandong Vigene Biosciences Co., Ltd., China). The final titre of each scAAV was 5.24 × 10^13^–8.01 × 10^13^ vg/mL.

### Stereotaxic surgery

Twenty-one-day-old female rats were stereotaxically injected with scAAVs. The rats were anaesthetised with 1% sodium pentobarbital (0.5 mL/100 g body weight). One microlitre of scAAV per side was microinjected bilaterally into the ARC (0.4 mm lateral, 1.6 mm posterior to bregma, 9.4 mm below the surface of the dura) at a rate of 250 nanolitres/min with a 10 μL Hamilton microsyringe^39^. The surgical procedure lasted ~20 min. Following surgery, the animals were placed in a clean cage on a heating pad until they returned to their home cages.

### IF staining analysis

Rats were weighed and delivered a fatal dose of 3% sodium pentobarbital (Sigma‒Aldrich, Germany) to induce euthanasia. To define the relative expression of FTO, m^6^A and PLCβ3, we performed IF staining using brain tissues. The hypothalamus was sectioned into 20 μm samples (at a range of bregma −1.72 mm to −4.36 mm). After washing with prechilled PBS five times for 5 min each, tissues were permeabilized using 0.2% Triton-100 for 5 min at room temperature and incubated with 3% sheep serum for 1 h. Then, tissues from female rats of the respective groups were incubated with primary antibodies, including anti-FTO (1:100, Abcam, USA), anti-m^6^A (1:100, SYSY, Germany) and anti-PLCβ3 (1:200, Santa Cruz, USA), in a refrigerated environment for 24 h. After washing with prechilled PBS 5 times for 5 min each, the sections were incubated with FITC-conjugated donkey anti-mouse IgG (H + L) (1:100), CoraLite594-conjugated donkey anti-rabbit IgG (H + L) (1:100) or Alexa Fluor 594-conjugated anti-mouse IgG (H + L) (1:500) for 1 h in a cool, dark place. After washing three times with prechilled PBS for 5 min, 800 mM 40,6-diamidino-2-phenylindole (DAPI) and an anti-fluorescence quencher were successively added to the sections. Then, the slides were covered with glass and sealed with nail polish. The stained tissues were captured with fluorescence microscopy (Leica, Germany).

### H&E staining

The ovaries, uterus and fat were isolated from female rats and fixed in 4% paraformaldehyde for 24 h. Subsequently, they were dehydrated in a series of ethanol concentrations, cleared in xylene, blocked in paraffin wax, and cut into serial 4 μm sections. The specimens were then stained with H&E. Representative visual fields were randomly selected for microphotography.

### Tissue collection

Rats were anaesthetised with 3% sodium pentobarbital (0.5 mL/100 g body weight), decapitated to collect the whole brain and subsequently frozen on dry ice. The hypothalami of female rats were dissected with four cuts. The anterior boundary was the anterior chiasma, the posterior boundary was the posterior margin of the body of the papilla, and the two sides were the temporal sulci. The fragments obtained had a thickness of approximately 2 mm. This fragment included the entire ARC.

### ELISA

Whole blood samples were collected from the hearts of female rats, and plasma was obtained after centrifugation. Then, the plasma and collected cell supernatant were stored at -80 °C until use. GnRH content from mice was then determined using ELISA kits (BIM, San Francisco, USA)^55^ and the supernatant, and the abundance of GnRH, LH, and FSH in the female rats’ serum was detected with other ELISA kits (Uscn Life Science Inc., Houston, USA) following the manufacturer’s instructions. The intra-assay variation was less than 10%, and the interassay variation was less than 15%. The experiments were conducted in triplicate.

### Calcium flux measurements

Calbryte 630 AM was purchased from AAT Bioquest, Inc. (USA) and dissolved in DMSO (Sigma‒Aldrich, Germany) as a transitional solvent. Cells were trypsinized and incubated with 250 μl of loading buffer containing 5 μM Calbryte 630 AM esters and 0.04% Pluronic F-127 (AAT Bioquest, USA) for 1 h at 37°C, according to the manufacturer’s instructions. Then, the cells were switched to Hanks and HEPES buffer and subjected to FACS analysis with an Accuri C6 Plus flow cytometer (BD) and fluorescence microscopy (Leica, Germany) to measure fluorescence intensity. The excitation and emission wavelengths were 640 nm and 660/20 nm, respectively. The results were further quantitatively analysed by FlowJo (v10) and ImageJ software.

### Dual-luciferase reporter and mutagenesis assays

*PLCβ3*-EXON with either wild-type or mutant (m^6^A replaced by T) was inserted downstream of pMIR-REPORT Luciferase. For the dual-luciferase reporter assay, cells seeded in 24-well plates were cotransfected with wild-type or mutant *PLCβ3*-EXON and OE-FTO (KD-FTO or empty vector). At 24 h posttransfection, the activity of firefly and Renilla luciferase in each well was determined with a Dual-Luciferase Reporter Assay System (DL101-01, Vazyme, China) according to the manufacturer’s protocol.

*PLCβ3*-EXON with wild-type m^6^A sites:

CTGAGTCAGT**GAACT**CCATCAGGAGGCTGGAAGAGGCCCAGAAGCAGCGCCACGAACGCCTCTTGGCAGGGCAACAGCAGGTCCTCCAGCAGCTAGTGGAAGAGGAGCCCAAGCTGGTGGCCCAGCTGACCCAGGAGTGTCAG**GAACA**GCGAGAGAGGCTGCCCCAGGAGATCCGTCGGTGCCTGCTGGGCG**AGACA**TCAGAGGGATTGGGGGATGGCCCCCTGGTGGCCTGTGCCAGCAATGGTCATGCAGCTGGGAGTGGTGGGCACCAGTCCGGCGCTGACTCGGAGAGCCAGGAGGAAAACACCCAGCTTTGAACTGGCGGAGCAAG

*PLCβ3*-EXON with mutant m^6^A sites:

CTGAGTCAGT**GATCT**CCATCAGGAGGCTGGAAGAGGCCCAGAAGCAGCGCCACGAACGCCTCTTGGCAGGGCAACAGCAGGTCCTCCAGCAGCTAGTGGAAGAGGAGCCCAAGCTGGTGGCCCAGCTGACCCAGGAGTGTCAG**GATCA**GCGAGAGAGGCTGCCCCAGGAGATCCGTCGGTGCCTGCTGGGCG**AGTCA**TCAGAGGGATTGGGGGATGGCCCCCTGGTGGCCTGTGCCAGCAATGGTCATGCAGCTGGGAGTGGTGGGCACCAGTCCGGCGCTGACTCGGAGAGCCAGGAGGAAAACACCCAGCTTTGAACTGGCGGAGCAAG.

### MeRIP-qPCR

An EpiQuik CUT&RUN m^6^A RNA Enrichment Kit (P-9018, EPIGENTEK, USA) was used to perform m^6^A immunoprecipitation in line with the manufacturer’s instructions. A HiScript III 1st Strand cDNA Synthesis Kit ( + gDNA Wiper) (R312, Vazyme, China) was used to perform reverse transcription and real-time qPCR. The relative expression of the m^6^A-modified target gene was determined as the Cq value of the m^6^A immunoprecipitate (IP) portion divided by the Cq value of the input portion.

### mRNA stability

GT1-7 cells with stable *FTO* gene knockdown and overexpression were cultured in 24-well plates until they reached 80-90% confluence. Then, the cells were treated with 5 μg/ml actinomycin D (A9415, Sigma, USA) and harvested at 0, 3, and 6 h. Total RNA was extracted using TRIzol, and 1 μg of total RNA was used for reverse transcription and qPCR.

### Statistics and reproducibility

GraphPad Prism 8.0 software (GraphPad, San Diego, CA, USA) and SPSS software (version 20; SPSS, Inc., Chicago IL, USA) were used for the statistical analysis. The results are presented as the mean ± standard error of the mean (SEM). The data were first subjected to normality and equal variance tests. The data that passed these two tests were then analysed by Student’s *t* test to compare two groups using SPSS. All results were analysed by one-way analysis of variance (ANOVA) with multiple comparisons, and Tukey’s multiple comparison test was applied. The number of samples per independent experiment were described in the legends. A *P* value of <0.05 was considered to indicate statistical significance.

### Reporting summary

Further information on research design is available in the Nature Portfolio Reporting Summary linked to this article.

### Supplementary information

Supplementary Information

Description of Additional Supplementary Files

Supplementary Data

Reporting Summary

## Data Availability

The raw sequence data reported in this paper have been deposited in the Genome Sequence Archive (Genomics, Proteomics & Bioinformatics 2021) in the National Genomics Data Center (Nucleic Acids Res 2022), China National Center for Bioinformation/Beijing Institute of Genomics, Chinese Academy of Sciences (GSA: CRA010515) and are publicly accessible at https://ngdc.cncb.ac.cn/gsa^56,57^. Numerical source data for all graphs in the manuscript can be found in supplementary data file. All the other data supporting the findings of this study are available within the article and its Supplemental Information files and from the corresponding author upon reasonable request. The source data behind the graphs in the paper is available in Supplementary Data.
