# Supplementary material for: FTO-mediated m6A demethylation regulates GnRH expression in the hypothalamus via the PLCβ3/Ca2+/CAMK signalling pathway
Source: Commun Biol. 2023 Dec 21;6:1297. doi: 10.1038/s42003-023-05677-2 (PMC10739951; doi:10.1038/s42003-023-05677-2)
Supplement: Supplementary file 1 — Supplementary Information [file 42003_2023_5677_MOESM1_ESM.pdf]

Supplementary Information

Zang et al.

FTO-mediated m^{6}A demethylation regulates GnRH expression in the hypothalamus via the PLCβ3/Ca^{2+}/CAMK signalling pathway

Supplementary Fig. 1

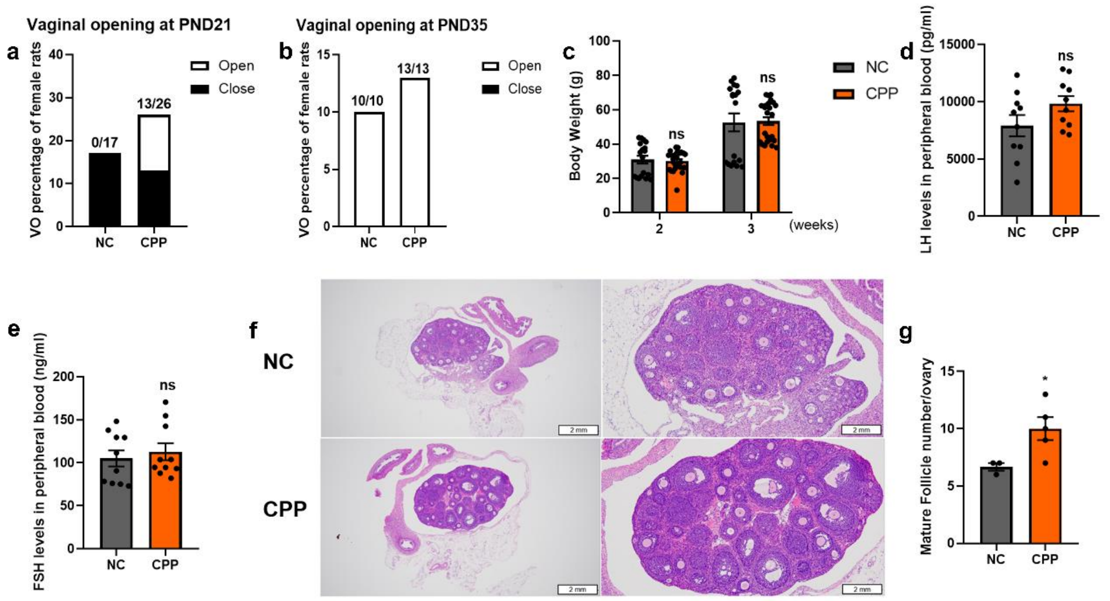

Supplementary Fig. 1. A female rat model of precocious puberty was successfully constructed. a, b VO percentage at 3 weeks (**a**) and 5 weeks (**b**) in NC and CPP female rats. **c** Body weights of female rats in the NC and CPP groups at 2 weeks and 3 weeks (n=18). **d, e** LH and FSH abundance in serum from 3-week-old female rats as determined by ELISA (NC, CPP; n = 10). **f** Ovary size and follicle morphology in female rats in the NC (n=3) and CPP (n=5) groups at 3 weeks with H&E staining. Bars, 2 mm. **g** Numbers of mature follicles in the ovaries of NC and CPP female rats

at 3 weeks. The bars represent the means ± SEMs. *$P < 0.05$, **$P < 0.01$, and ***$P < 0.001$ versus the NC group by Student's t test.

Supplementary Fig. 2

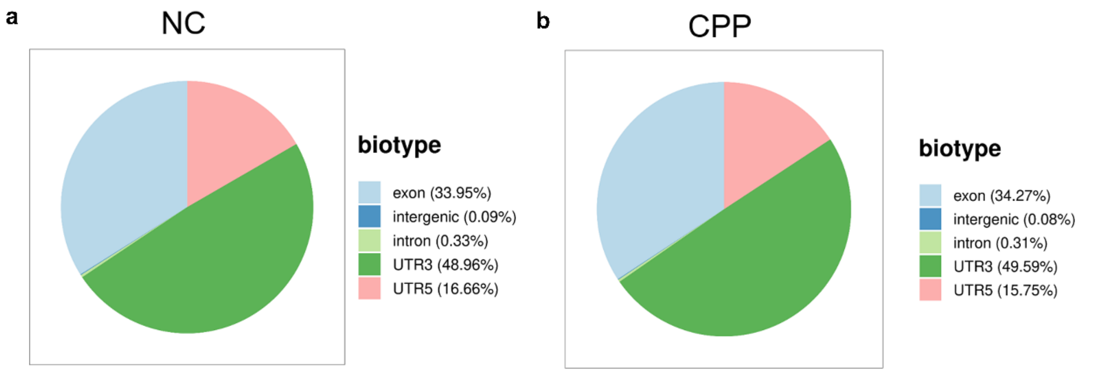

Supplementary Fig. 2 Distribution of m^{6}A peaks in the NC and CPP female rats. a, b Proportion of m^{6}A peak distribution in the exon, intergenic, intron, 3'UTR or 5'UTR across the entire set of mRNA transcripts between the NC (**a**) and CPP (**b**) female rats.

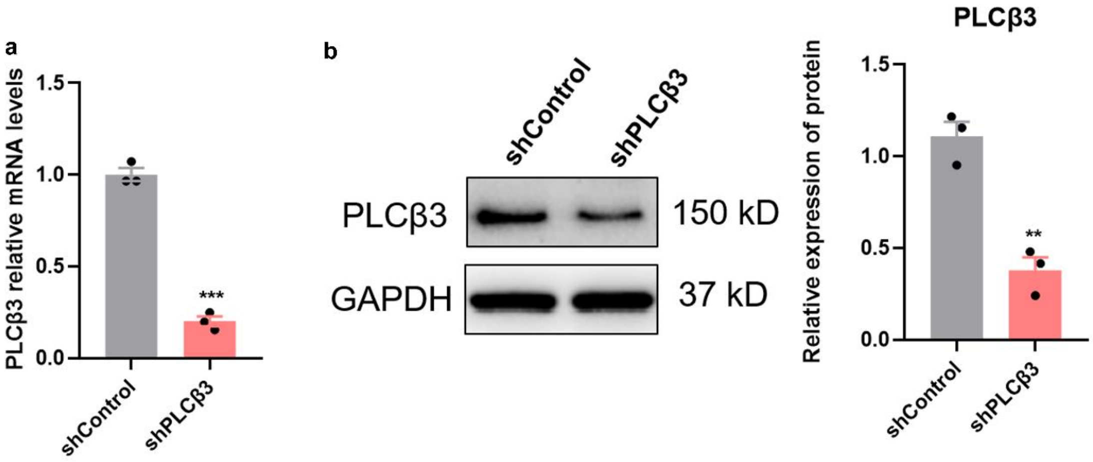

Supplementary Fig. 3 A PLCβ3 silencing plasmid was successfully constructed. a mRNA expression of *PLCβ3* in control and PLCβ3-knockdown cells as determined by

qPCR (n=3). **b** PLCβ3 protein concentration as determined by western blotting (n=3). The bars represent the means ± SEMs. **$P < 0.01$ and ***$P < 0.001$ versus the sh-control group by Student's t test.

Supplementary Fig. 4

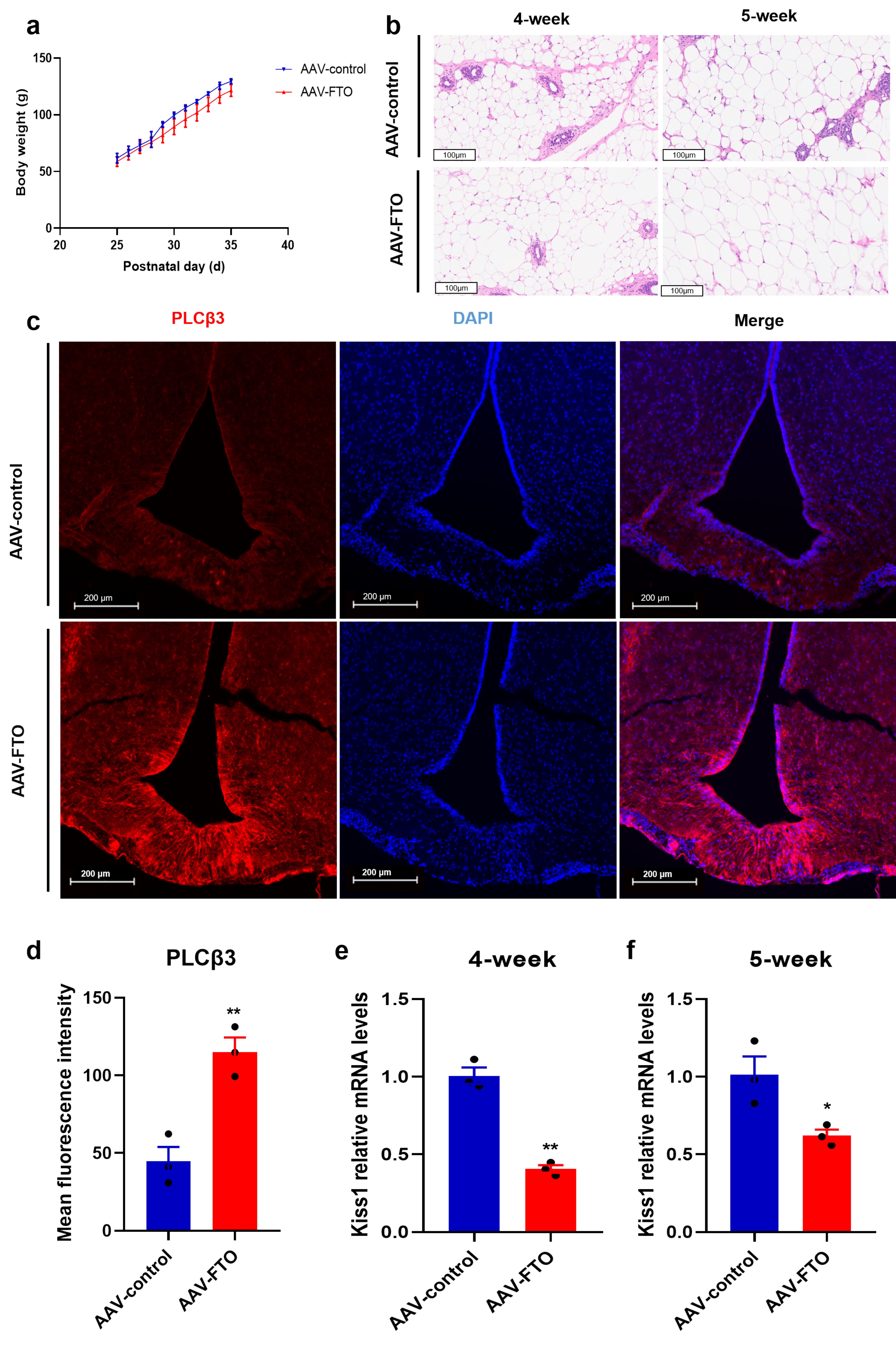
a
Body weight (g)
150
100
50
0
20
25
30
35
40
Postnatal day (d)
AAV-control
AAV-FTO
b
4-week
5-week
AAV-control
AAV-FTO
100μm
c
PLCβ3
DAPI
Merge
AAV-control
AAV-FTO
200 μm
d
PLCβ3
Mean fluorescence intensity
**
e
4-week
Kiss1 relative mRNA levels
1.5
1.0
0.5
0.0
**
f
5-week
*
AAV-control
AAV-FTO

Supplementary Fig. 4 Effects of intra-ARC AAV-FTO and AAV-control administration on female rats at 4 weeks and 5 weeks. Three-week-old female rats were microinjected with AAV-FTO or AAV-control in the ARC. a Body weights of female rats receiving AAV transfection beginning at postnatal day 25 (PND25) (n=6). **b** Pathological assessment of fat by H&E staining in the ARC-infected group at 4 weeks and 5 weeks (n=6). Scale bars, 100 μm. **c** Abundance of PLCβ3 (red colour) and DAPI (blue colour) in the ARC as determined by IF (NC, CPP; n = 3). Scale bars, 200 μm. **d** Mean fluorescence intensity of PLCβ3 as calculated by ImageJ software. **e, f** *Kiss1* mRNA levels detected by qPCR in the ARC in 4-week (**e**) or 5-week (**f**) female rats receiving AAV-control (n = 3) or AAV-FTO (n = 3). AAV-FTO for the FTO-overexpression group, AAV-control for the AAV negative control group. The bars represent the means ± SEMs. *$P < 0.05$ and **$P < 0.01$ versus the AAV-control group by Student's t test.

Supplementary Table 1. Sequences of qPCR primers.

Gene	Species	Forward primer (5'-3')	Reverse primer (5'-3')
**qPCR primers**			
β-actin	mouse	AAGATCAAGATCATTGCTCCTCC	GACTCATCGTACTCCTGCTTGC
FTO	mouse	GAGCAGCCTACAACGTGACT	GAAGCTGGACTCGTCCTCAC
GnRH	mouse	TGATCCTCAAACTGATGGCCG	CGCAACCCATAGGACCAGTG
CAM	mouse	GGCTGACCAACTGACTGA	TTACCATCCGCATCTACT

PLCβ3	mouse	CGAGACTCAACGAAGTGCTG	ACCTCCTCCCCATTGCTTAG
β-actin	rat	TGCCGCATCCTCTTCCTC	GGTCTTTACGGATGTCAACG
GnRH	rat	CCGCTGTTGTTCTGTTGACTGTG	GGGGTTCTGCCATTTGATCCTC
Kiss1	rat	AGCTGCTGCTTCTCCTCTGT	AGGCTTGCTCTCTGCATACC
FTO	rat	GACACTTGGCTTCCTTACCTG	CTCACCACGTCCCGAAACAA
CAM	rat	CGACTTCCCTGAATTCCTGA	TCTGCTGCACTGATGTAGCC
CAMKII	rat	AAGATGTGCGACCCTGGAATG	TGTAGGCGATGCAGGCTGAC
ALKBH5	rat	CGCGGTCATCAACGACTACC	ATGGGCTTGAACTGGAACTTG
METTL3	rat	CTGGGCACTTGGATTTAAGGAA	TGAGAGGTGGTGTAGCAACTT
METTL14	rat	GAGCTGAGAGTGCGGATAGC	GCAGATGTATCATAGGAAGCCC
FMR1	rat	CAATGGCGCTTTCTACAAGGC	TCTGGTTGCCAGTTGTTTTCA
METTL16	rat	GACAAACCACCTGACTTCGCA	TCTGACTGCTTCGGGGTCTT
RBM15	rat	CGAGTCCGCTGTGTGAAAC	TCCCCACGAGAACTGGAGTC
RBMX	rat	AGAGACGAATGAGAAAGCCCT	AGTGACAAAAGCGAATCCTCTTG
VIRMA	rat	GGTTCGTTTTCCGTGTGTGG	GCCACTATGGGCTCGTACTC
WTAP	rat	GAACCTCTTCCTAAAAAGGTCCG	TTAACTCATCCCGTGCCATAAC
YTHDC1	rat	GTCCACATTGCCTGTAAATGAGA	GGAAGCACCCAGTGTATAGGA
YTHDC2	rat	GAAGATCGCCGTCAACATCG	GCTCTTTCCGTACTGGTCAAA
YTHDF1	rat	ACAGTTACCCCTCGATGAGTG	GGTAGTGAGATACGGGATGGGA
YTHDF2	rat	GAGCAGAGACCAAAAGGTCAAG	CTGTGGGCTCAAGTAAGGTTC
YTHDF3	rat	GATCAGCCTATGCCATATCTGAC	CCCCTGGTTGACTAAAAACACC

MeRIP-qPCR primers

PLCβ3 peak	mouse	GTCAGGAACAGCGAGAGAGG	CCAGTTCAAAGCTGGGTGTT

Supplementary Table 2 The information of antibodies used in this study.

**Antibodies**	**Source**	**Identifier**	**Host**	**Application**
GAPDH	CST	#2118	Rabbit	WB
FTO	ABclonal	A1438	Rabbit	WB
FTO	Abcam	Ab92821	mouse	IF
PLCβ3	Santa Cruz	sc-133231	mouse	IF
m6A	SYSY	202003	Rabbit	IF
PLCβ3	CST	#14247	Rabbit	WB
CAM	ABclonal	A4885	Rabbit	WB
CAMKII	CST	#4436	Rabbit	WB
p-CAMKII	CST	#12716	Rabbit	WB
HRP Goat Anti-Rabbit IgG (H+L)	ABclonal	AS014	Goat	WB
FITC-conjugated donkey anti-mouse IgG (H+L)	Proteintech	SA00003-9	Donkey	IF
CoraLite594-conjugated donkey anti-Rabbit IgG (H+L)	Proteintech	SA00013-8	Donkey	IF

Alexa Fluor 594-conjugated goat anti-mouse IgG(H+L)	CST	#8890	Goat	IF

Supplementary Fig. 5

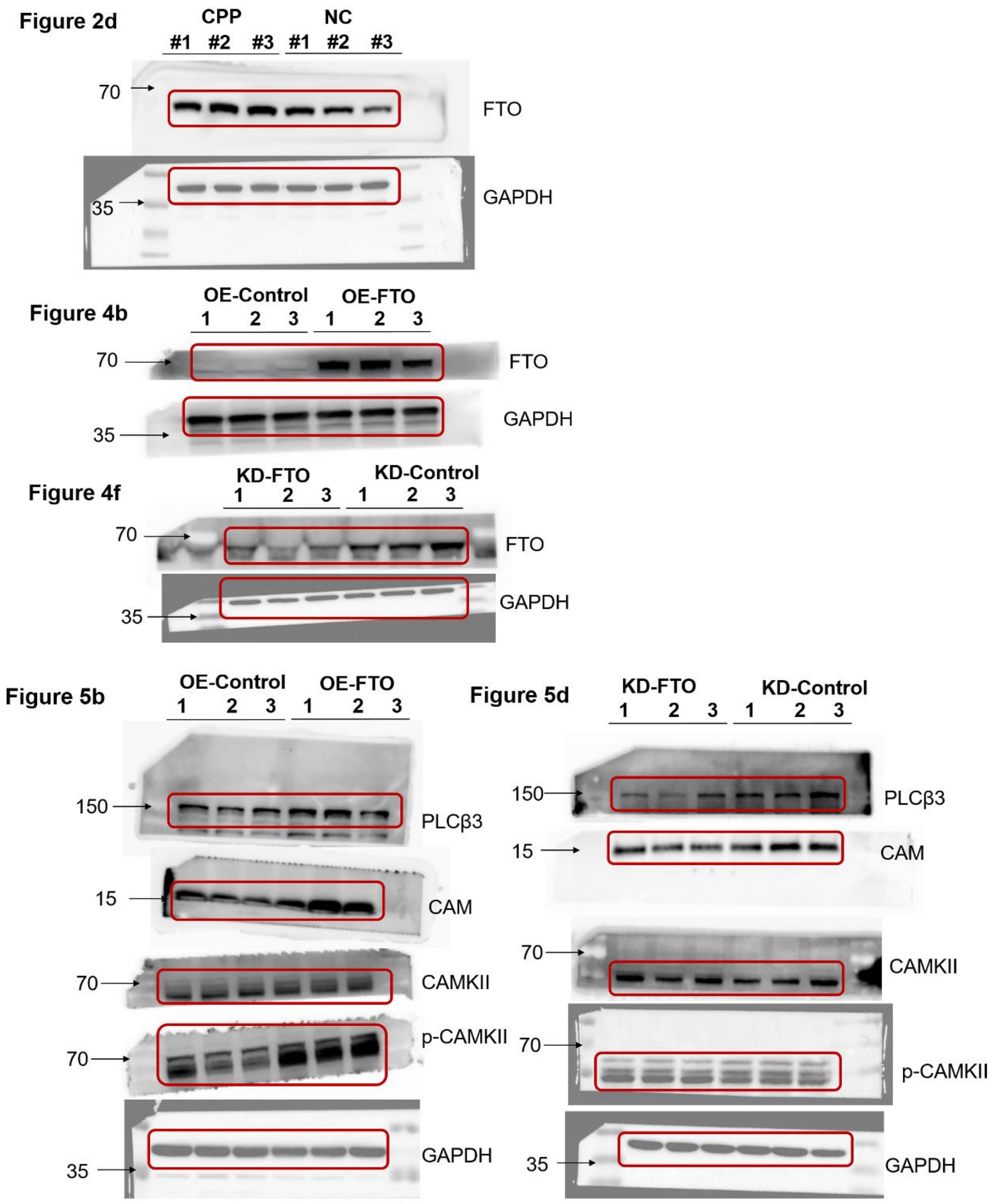

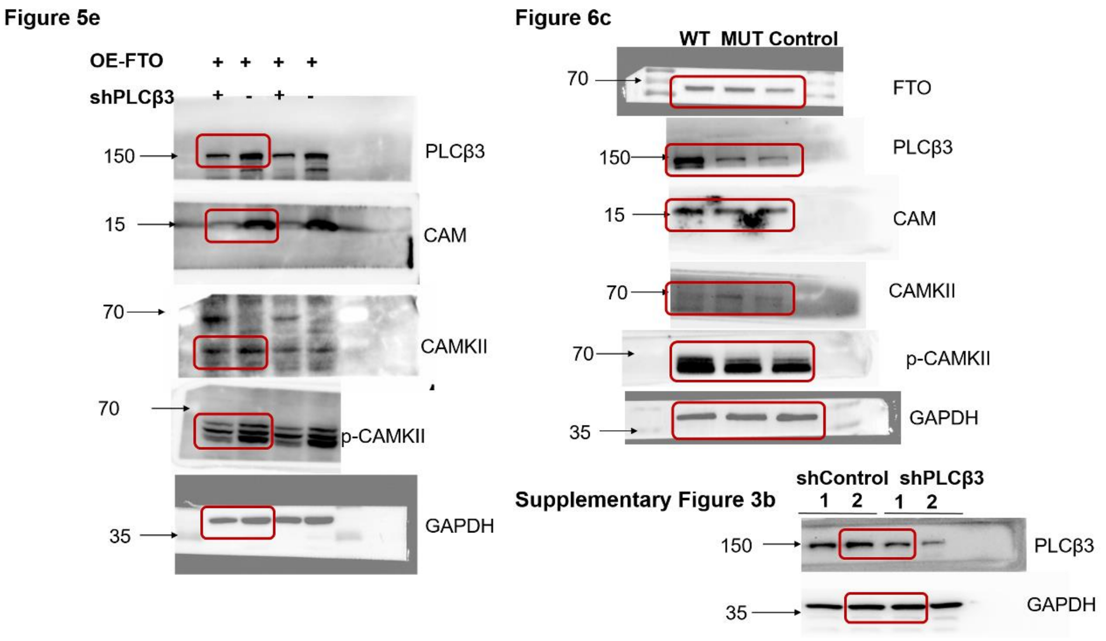

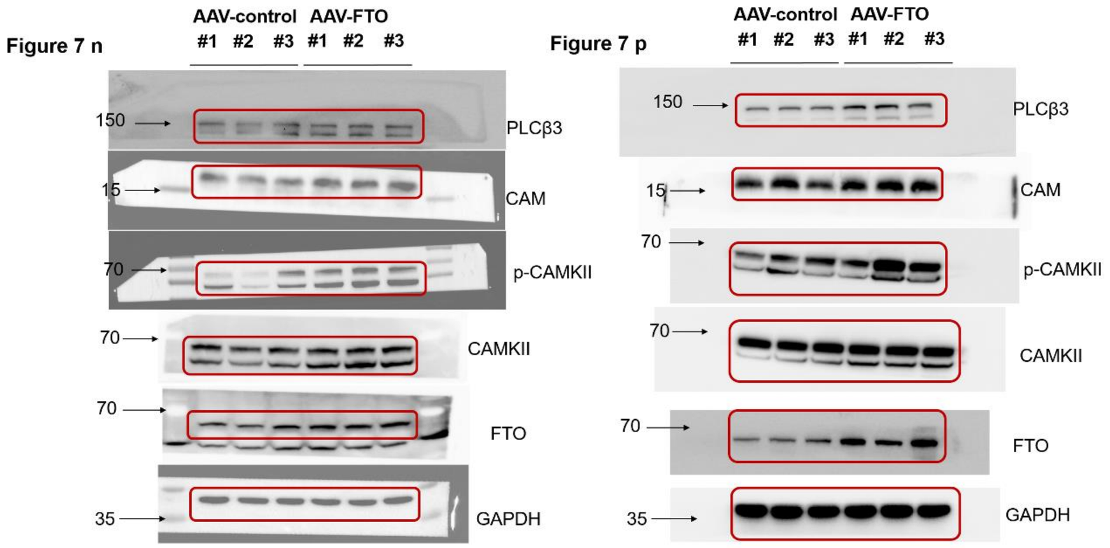

Supplementary Fig. 5 Unedited and uncropped Western blots of all the indicated figures in main figures and supplementary figures.
