# Supplementary material for: FTO-mediated m6A demethylation regulates GnRH expression in the hypothalamus via the PLCβ3/Ca2+/CAMK signalling pathway
Source: Commun Biol. 2023 Dec 21;6:1297. doi: 10.1038/s42003-023-05677-2 (PMC10739951; doi:10.1038/s42003-023-05677-2)
Supplement: Supplementary file 4 — Reporting Summary [file 42003_2023_5677_MOESM4_ESM.pdf]

Corresponding author(s): Xiaoqin Yin and Pin Li

Last updated by author(s): Nov 21, 2023

Reporting Summary

Nature Portfolio wishes to improve the reproducibility of the work that we publish. This form provides structure for consistency and transparency in reporting. For further information on Nature Portfolio policies, see our [Editorial Policies] and the [Editorial Policy Checklist].

Statistics

For all statistical analyses, confirm that the following items are present in the figure legend, table legend, main text, or Methods section.

n/a	Confirmed	
☐	☒	The exact sample size (*n*) for each experimental group/condition, given as a discrete number and unit of measurement
☐	☒	A statement on whether measurements were taken from distinct samples or whether the same sample was measured repeatedly
☐	☒	The statistical test(s) used AND whether they are one- or two-sided<br>*Only common tests should be described solely by name; describe more complex techniques in the Methods section.*
☒	☐	A description of all covariates tested
☐	☒	A description of any assumptions or corrections, such as tests of normality and adjustment for multiple comparisons
☐	☒	A full description of the statistical parameters including central tendency (e.g. means) or other basic estimates (e.g. regression coefficient) AND variation (e.g. standard deviation) or associated estimates of uncertainty (e.g. confidence intervals)
☐	☒	For null hypothesis testing, the test statistic (e.g. *F*, *t*, *r*) with confidence intervals, effect sizes, degrees of freedom and *P* value noted<br>*Give P values as exact values whenever suitable.*
☒	☐	For Bayesian analysis, information on the choice of priors and Markov chain Monte Carlo settings
☒	☐	For hierarchical and complex designs, identification of the appropriate level for tests and full reporting of outcomes
☒	☐	Estimates of effect sizes (e.g. Cohen's *d*, Pearson's *r*), indicating how they were calculated

Our web collection on statistics for biologists contains articles on many of the points above.

Software and code

Policy information about availability of computer code

Data collection	AccuriTM C6 Plus flow cytometer (BD) was used to detect free ca2+ abundance; Fluorescence microscopy (Leica, Germany) was used to observe targets expression and location.
Data analysis	SPSS (version 20) and GraphPad Prism (version 8.0) softwares were used for statistical analysis; Image J (version1.51) was used for western blotting and fluorescence intensity analysis; FlowJo (version 10) was used for ca2+ expression; Quality control was conducted using FASTQC (version 0.11.5); Motif analysis was conducted using IGV (version 2.14.1).

For manuscripts utilizing custom algorithms or software that are central to the research but not yet described in published literature, software must be made available to editors and reviewers. We strongly encourage code deposition in a community repository (e.g. GitHub). See the Nature Portfolio guidelines for submitting code & software for further information.

Data

Policy information about availability of data

All manuscripts must include a data availability statement. This statement should provide the following information, where applicable:

- Accession codes, unique identifiers, or web links for publicly available datasets
- A description of any restrictions on data availability
- For clinical datasets or third party data, please ensure that the statement adheres to our policy

Raw sequencing data is deposited to the public data repository (Genome Sequence Archive), and the accession ID is included in Data availability statement. All the

other data supporting the findings of this study are available within the article and its Supplementary Information files and from the corresponding author upon reasonable request.

Human research participants

Policy information about studies involving human research participants and Sex and Gender in Research.

Reporting on sex and gender	Not applicable
Population characteristics	Not applicable
Recruitment	Not applicable
Ethics oversight	Not applicable

Note that full information on the approval of the study protocol must also be provided in the manuscript.

Field-specific reporting

Please select the one below that is the best fit for your research. If you are not sure, read the appropriate sections before making your selection.

☒ Life sciences ☐ Behavioural & social sciences ☐ Ecological, evolutionary & environmental sciences

For a reference copy of the document with all sections, see nature.com/documents/nr-reporting-summary-flat.pdf

Life sciences study design

All studies must disclose on these points even when the disclosure is negative.

Sample size	No statistical methods were applied to determine the sample size, the sample size are similar to those reported in previous literature.
Data exclusions	No data were excluded.
Replication	All experiments were performed at least two replicates.
Randomization	The Sprague-Dawley rats used in all experiments were assigned randomly to either control or experimental groups.
Blinding	All behavioral data analysis in this study were carried out blind to prevent any bias.

Reporting for specific materials, systems and methods

We require information from authors about some types of materials, experimental systems and methods used in many studies. Here, indicate whether each material, system or method listed is relevant to your study. If you are not sure if a list item applies to your research, read the appropriate section before selecting a response.

Materials & experimental systems

n/a	Involved in the study	
☐	☒	Antibodies
☐	☒	Eukaryotic cell lines
☒	☐	Palaeontology and archaeology
☐	☒	Animals and other organisms
☒	☐	Clinical data
☒	☐	Dual use research of concern

Methods

n/a	Involved in the study	
☒	☐	ChIP-seq
☐	☒	Flow cytometry
☒	☐	MRI-based neuroimaging

Antibodies

Antibodies used	Antibodies for western blotting: anti-FTO (ABclonal, A1438, 1:1000), anti-CAM (ABclonal, A4885, 1:1000), anti-CAMKII (Cell Signaling Technology, #4436, 1:1000); anti-Phospho-CAMKII (Cell Signaling Technology, #12716, 1:1000); anti-PLCβ3 (Cell Signaling Technology, #14247, 1:1000); anti-GAPDH (Cell Signaling Technology, #2118, 1:1000); anti-rabbit (ABclonal, AS014, 1:5000); antibodies for m6A immunofluorescence: anti-m6A antidody (Synaptic System, 202003, 1:100); antibodies for FTO immunofluorescence: anti-FTO (Abcam, Ab92821, 1:100); anti-PLCβ3 (Santacruz, sc-133231, 1:200);anti-mouse for immunofluorescence: FITC-conjugated donkey anti-mouse IgG (H+L) (Proteintech, SA00003-9, 1:100),Alexa Fluor 594-conjugated

	goat anti-mouse IgG(H+L) (CST, #8890, 1:500) ; anti-rabbit for immunofluorescence: CoraLite594-conjugated donkey anti-Rabbit IgG (H+L) (Proteintech, SA00013-8, 1:100); antibodies for MeRIP-Seq :(Synaptic System, 202003, 1:1000); antibodies for gene-specific m6A qPCR :anti-m6A antibody ((Synaptic System, 202003, 1:1000))
Validation	All antibodies used in out study were validation on the manufacturer's website.<br>anti-FTO (ABclonal, A1438)https://abclonal.com.cn/catalog/A1438<br>anti-CAM (ABclonal, A4885)https://abclonal.com.cn/catalog/A4885<br>anti-CAMKII (Cell Signaling Technology, #4436)https://www.cellsignal.cn/products/primary-antibodies/camkii-pan-d11a10-rabbit-mab/4436?site-search-type=Products&N=4294956287&Ntt=4436&fromPage=plp&_requestid=1928953<br>anti-Phospho-CAMKII (Cell Signaling Technology, #12716)https://www.cellsignal.cn/products/primary-antibodies/phospho-camkii-thr286-d21e4-rabbit-mab/12716?site-search-type=Products&N=4294956287&Ntt=12716&fromPage=plp&_requestid=1929060<br>anti-PLCβ3 (Cell Signaling Technology, #14247)https://www.cellsignal.cn/products/primary-antibodies/plcbeta3-d9d6s-rabbit-mab/14247?site-search-type=Products&N=4294956287&Ntt=14247%29&fromPage=plp&_requestid=1929116<br>anti-GAPDH (Cell Signaling Technology, #2118)https://www.cellsignal.cn/products/primary-antibodies/gapdh-14c10-rabbit-mab/2118?site-search-type=Products&N=4294956287&Ntt=2118&fromPage=plp&_requestid=1929169<br>anti-rabbit (ABclonal, AS014) https://abclonal.com.cn/catalog/AS014<br>anti-m6A antibody (Synaptic System, 202003)https://www.sysy.com/product/202003#list<br>anti-FTO(Abcam, Ab92821)https://www.abcam.cn/products/primary-antibodies/fto-antibody-5-2h10-ab92821.html<br>anti-PLCβ3 (Santacruz, sc-133231)https://www.scbt.com/zh/p/plc-beta3-antibody-d-7?requestFrom=search<br>FITC-conjugated donkey anti-mouse IgG (H+L) (Proteintech, SA00003-9)https://www.ptgcn.com/products/Fluorescein-FITC-conjugated-Affinipure-Donkey-Anti-Mouse-IgG-H-L.htm<br>Alexa Fluor 594-conjugated goat anti-mouse IgG(H+L) (CST, #8890) https://www.cellsignal.com/products/secondary-antibodies/anti-mouse-igg-h-l-f-ab-2-fragment-alexa-fluor-594-conjugate/8890<br>CoraLite594-conjugated donkey anti-Rabbit IgG (H+L) (Proteintech, SA00013-8)https://www.ptgcn.com/products/CoraLite594-conjugated-Donkey-Anti-Rabbit-IgG-H-L.htm

Eukaryotic cell lines

Policy information about cell lines and Sex and Gender in Research

Cell line source(s)	the American Type Culture Collection (ATCC, Manassas, VA)
Authentication	No
Mycoplasma contamination	Cells were routinely tested for mycoplasma contamination using MycoAlert Mycoplasma Detection Kit.
Commonly misidentified lines<br>(See ICLAC register)	No

Animals and other research organisms

Policy information about studies involving animals; ARRIVE guidelines recommended for reporting animal research, and Sex and Gender in Research

Laboratory animals	Sprague-Dawley rat
Wild animals	No
Reporting on sex	In this study, only female rats were applied, and the sex was considered in study design. The annual incidence of CPP is increasing gradually, and girls are 5-10 times more likely to develop CPP than boys. Due to this phenomenon, we only select female rats to investigate the mechanism of disease.
Field-collected samples	Sprague-Dawley female rats were obtained from Shanghai Jie Sijie Experimental Animal Co., Ltd. and housed under a 12h/12h light/dark cycle at a controlled temperature.
Ethics oversight	This animal experiment was authorized by the Animal Experiment Committee of Shanghai Children's Hospital, School of medicine, Shanghai Jiao Tong University.

Note that full information on the approval of the study protocol must also be provided in the manuscript.

Flow Cytometry

Plots

Confirm that:

☒ The axis labels state the marker and fluorochrome used (e.g. CD4-FITC).

☒ The axis scales are clearly visible. Include numbers along axes only for bottom left plot of group (a 'group' is an analysis of identical markers).

☐ All plots are contour plots with outliers or pseudocolor plots.

☒ A numerical value for number of cells or percentage (with statistics) is provided.

Methodology

Sample preparation	The cells were trypsinized and incubated with 250 µl loading buffer containing 5 µM CalbryteTM 630 AM esters and 0.04% Pluronic F-127 (AAT Bioquest, USA) for 1 h at 37?. Then, cells were switched to Hanks and Hepes buffer and subjected to FACS analysis.
Instrument	AccuriTM C6 Plus flow cytometer (BD)
Software	FlowJo (version 10) software
Cell population abundance	Flow Cytometry results were ultimately presented in terms of fluorescence intensity rather than cell population proportion.
Gating strategy	The SSC-A/FSC-A gate was preliminarily established in the cells, and the cluster of cells with positive results was named L1. The L1 cell population was further set as FSC-H/FSC-A gate, and the positive cells were named as Single Cells. The Single Cells population was further set into the SSC-A/calium-APC-A gate, with positive cells as the target cells.

☒ Tick this box to confirm that a figure exemplifying the gating strategy is provided in the Supplementary Information.
